# Increased soluble urokinase plasminogen activator levels modulate monocyte function to promote atherosclerosis

**DOI:** 10.1172/JCI158788

**Published:** 2022-12-15

**Authors:** George Hindy, Daniel J. Tyrrell, Alexi Vasbinder, Changli Wei, Feriel Presswalla, Hui Wang, Pennelope Blakely, Ayse Bilge Ozel, Sarah Graham, Grace H. Holton, Joseph Dowsett, Akl C. Fahed, Kingsley-Michael Amadi, Grace K. Erne, Annika Tekmulla, Anis Ismail, Christopher Launius, Nona Sotoodehnia, James S. Pankow, Lise Wegner Thørner, Christian Erikstrup, Ole Birger Pedersen, Karina Banasik, Søren Brunak, Henrik Ullum, Jesper Eugen-Olsen, Sisse Rye Ostrowski, on behalf of the DBDS Consortium, Mary E. Haas, Jonas B. Nielsen, Luca A. Lotta, on behalf of the Regeneron Genetics Center, Gunnar Engström, Olle Melander, Marju Orho-Melander, Lili Zhao, Venkatesh L. Murthy, David J. Pinsky, Cristen J. Willer, Susan R. Heckbert, Jochen Reiser, Daniel R. Goldstein, Karl C. Desch, Salim S. Hayek

**Affiliations:** 1 Department of Clinical Sciences, Lund University, Malmö, Sweden.; 2 Department of Population Medicine, Qatar University College of Medicine, QU Health, Doha, Qatar.; 3 Program in Medical and Population Genetics, Broad Institute of MIT and Harvard, Cambridge, Massachusetts, USA.; 4 Division of Cardiology, Department of Internal Medicine, University of Michigan, Ann Arbor, Michigan, USA.; 5 Department of Medicine, Rush University Medical Center, Chicago, Illinois, USA.; 6 Department of Human Genetics, University of Michigan, Ann Arbor, Michigan, USA.; 7 Department of Clinical Immunology, Copenhagen University Hospital, Copenhagen, Denmark.; 8 Cardiovascular Health Research Unit, Division of Cardiology, University of Washington, Seattle, Washington, USA.; 9 Division of Epidemiology and Community Health, University of Minnesota, Minneapolis, Minnesota, USA.; 10 Department of Immunology, Aarhus University Hospital, Aarhus, Denmark.; 11 Department of Immunology, Zealand University Hospital, Køge, Denmark.; 12 Novo Nordisk Foundation Center for Protein Research, Faculty of Health and Medical Sciences, University of Copenhagen, Copenhagen, Denmark.; 13 State Serum Institute, Copenhagen, Denmark.; 14 Department of Clinical Research, Copenhagen University Hospital Amager and Hvidovre, Hvidovre, Denmark.; 15 Members of the Danish Blood Donor Study (DBDS) Consortium are detailed in Supplemental Acknowledgments.; 16 Regeneron Genetics Center, Regeneron Pharmaceuticals Inc., Tarrytown, New York, USA.; 17 Members of the Regeneron Genetics Center are detailed in Supplemental Acknowledgments.; 18 Department of Biostatistics, School of Public Health, University of Michigan, Ann Arbor, Michigan, USA.; 19 Department of Epidemiology, University of Washington, Seattle, Washington, USA.; 20 Division of Neonatal-Perinatal Medicine, Department of Pediatrics, Program in Cellular and Molecular Biology, University of Michigan, Ann Arbor, Michigan, USA.

**Keywords:** Cardiology, Atherosclerosis, Innate immunity

## Abstract

People with kidney disease are disproportionately affected by atherosclerosis for unclear reasons. Soluble urokinase plasminogen activator receptor (suPAR) is an immune-derived mediator of kidney disease, levels of which are strongly associated with cardiovascular outcomes. We assessed suPAR’s pathogenic involvement in atherosclerosis using epidemiologic, genetic, and experimental approaches. We found serum suPAR levels to be predictive of coronary artery calcification and cardiovascular events in 5,406 participants without known coronary disease. In a genome-wide association meta-analysis including over 25,000 individuals, we identified a missense variant in the plasminogen activator, urokinase receptor (*PLAUR*) gene (rs4760), confirmed experimentally to lead to higher suPAR levels. Mendelian randomization analysis in the UK Biobank using rs4760 indicated a causal association between genetically predicted suPAR levels and atherosclerotic phenotypes. In an experimental model of atherosclerosis, proprotein convertase subtilisin/kexin–9 (*Pcsk9*) transfection in mice overexpressing suPAR (*suPAR^Tg^*) led to substantially increased atherosclerotic plaques with necrotic cores and macrophage infiltration compared with those in WT mice, despite similar cholesterol levels. Prior to induction of atherosclerosis, aortas of *suPAR^Tg^* mice excreted higher levels of CCL2 and had higher monocyte counts compared with WT aortas. Aortic and circulating *suPAR^Tg^* monocytes exhibited a proinflammatory profile and enhanced chemotaxis. These findings characterize suPAR as a pathogenic factor for atherosclerosis acting at least partially through modulation of monocyte function.

## Introduction

People with chronic kidney disease (CKD) are disproportionately affected by cardiovascular disease (CVD), with two-thirds of patients having at least one form of CVD, atherosclerosis being the most common (1). Conversely, over 40% of patients with CVD have signs of kidney disease (1). The reasons for which a large proportion of patients with CKD have concomitant CVD are unknown. Little progress has been made in understanding the nontraditional contributors of CKD to CVD risk, and the development of therapies targeting purported mechanisms such as vitamin D deficiency, hyperuricemia, and hyperphosphatemia have largely been ineffective (2).

Soluble urokinase plasminogen activator receptor (suPAR) is the circulating form of uPAR, a glycosyl-phosphatidylinositol–anchored (GPI-anchored) 3-domain (DI, DII, and DIII) receptor protein encoded by the plasminogen activator, urokinase receptor (*PLAUR*) gene. The membrane-bound form, uPAR, is expressed on a variety of cells, including immune cells, endothelial cells, and podocytes, with levels in circulation thought to reflect the aggregate activity of the uPAR system: immune activity, proteolysis, and extracellular matrix remodeling (3–9). suPAR has gained notoriety as an immune-derived pathogenic factor and therapeutic target for kidney disease (10–18). Blood suPAR levels are strongly predictive of incident kidney disease in different patient populations (11, 12, 14–16, 18, 19), and transgenic mice overexpressing suPAR exhibit phenotypes of kidney disease (8, 10, 17, 20). Most importantly, interfering with the suPAR pathway through bone marrow ablation (10), anti-suPAR monoclonal antibodies (8, 18), or small molecule inhibitors of suPAR can prevent and reverse kidney injury in experimental models, strongly supporting a pathogenic role for suPAR (21).

suPAR also appears to have an important role in CVD. Its levels are tightly correlated with the most prominent risk factors for atherosclerosis: smoking, diabetes mellitus, and CKD (14, 22, 23). suPAR levels are elevated within atherosclerotic plaque and correlate with intraplaque proinflammatory cytokines, including monocyte chemoattractant protein 1, TNF-α, IL-1β, and IL-66 (24). Patients with peripheral arterial disease have very high plasma suPAR levels that correlate positively with the number of clinically significant atherosclerotic sites and are predictive of vascular events (25). suPAR levels have been consistently associated with incident CVD and poor outcomes in various patient groups, including critically ill patients, those with HIV, cancer, and kidney disease, as well as the general population (24, 26–34). The association between suPAR and cardiovascular outcomes is independent of its impact on the kidneys, as even patients with end-stage renal disease can be risk stratified using suPAR (35).

Thus, suPAR is an immune-derived pathogenic factor for kidney disease and a strong biomarker of CVD, notably atherosclerosis. Here, we used epidemiologic, genetic, and experimental approaches to assess whether suPAR is causally involved in atherosclerosis. We first examined the relationship between suPAR levels and coronary artery calcifications (CAC) — a surrogate of atherosclerosis — and CVD events in 5,406 participants without known preexisting CVD. We sought genetic evidence of a causal role for suPAR in CVD by performing a genome-wide association study (GWAS) meta-analysis for suPAR levels in over 24,000 individuals, confirming experimentally a missense variant that specifically led to higher suPAR levels and using Mendelian randomization (MR) and rare variant association to leverage the genotypes and disease phenotypes in 500,000 participants of the UK Biobank (https://www.ukbiobank.ac.uk/). Finally, using a well-established murine model of atherosclerosis, we assessed whether mice overexpressing suPAR were more prone to atherosclerotic disease compared with WT mice and examined the impact of suPAR on monocyte profile and function.

## Results

### suPAR levels associated cross-sectionally with CAC and predicted CAC progression in the Multi-Ethnic Study of Atherosclerosis

#### Study cohort characteristics.

Overall, Multi-Ethnic Study of Atherosclerosis (MESA) participants in this analysis had a mean (SD) age of 63 (10) years, 48.5% were male, 51.5% were female, and 28%, 11%, 22%, and 38% were Black, Chinese American, Hispanic American, and White, respectively. The median baseline suPAR level was 2.5 (IQR 2.0, 3.1) ng/mL. With increasing suPAR categories, participants were older, consisted of a larger proportion of women, and had a history of smoking, diabetes, and hypertension (*P* < 0.001 for all) (Supplemental Table 1; supplemental material available online with this article; https://doi.org/10.1172/JCI158788DS1). The median CAC for the cohort was 1.91 Agatston units (AU), and a total of 2,636 (48.8%) participants had a CAC greater than 0.

#### Association between baseline CAC scores and suPAR.

Participants with CAC greater than 0 had significantly higher suPAR levels than those with CAC equal to 0 (2.56 ng/mL, IQR 2.05–3.23, compared with 2.34 ng/mL, IQR 1.89–2.95, respectively, *P* < 0.001). Baseline suPAR levels correlated modestly with baseline CAC (Spearman’s rank *r* = 0.14, *P* < 0.001). After adjustment for demographics, cardiac risk factors, and laboratory data, higher baseline suPAR levels were significantly associated with a higher CAC score at baseline: for a 2-fold difference in suPAR levels, baseline CAC scores were higher on average by 28.7 AU (95% CI 8.0–49.5) (Supplemental Table 2).

#### Baseline suPAR and longitudinal CAC.

**Figure 1:**
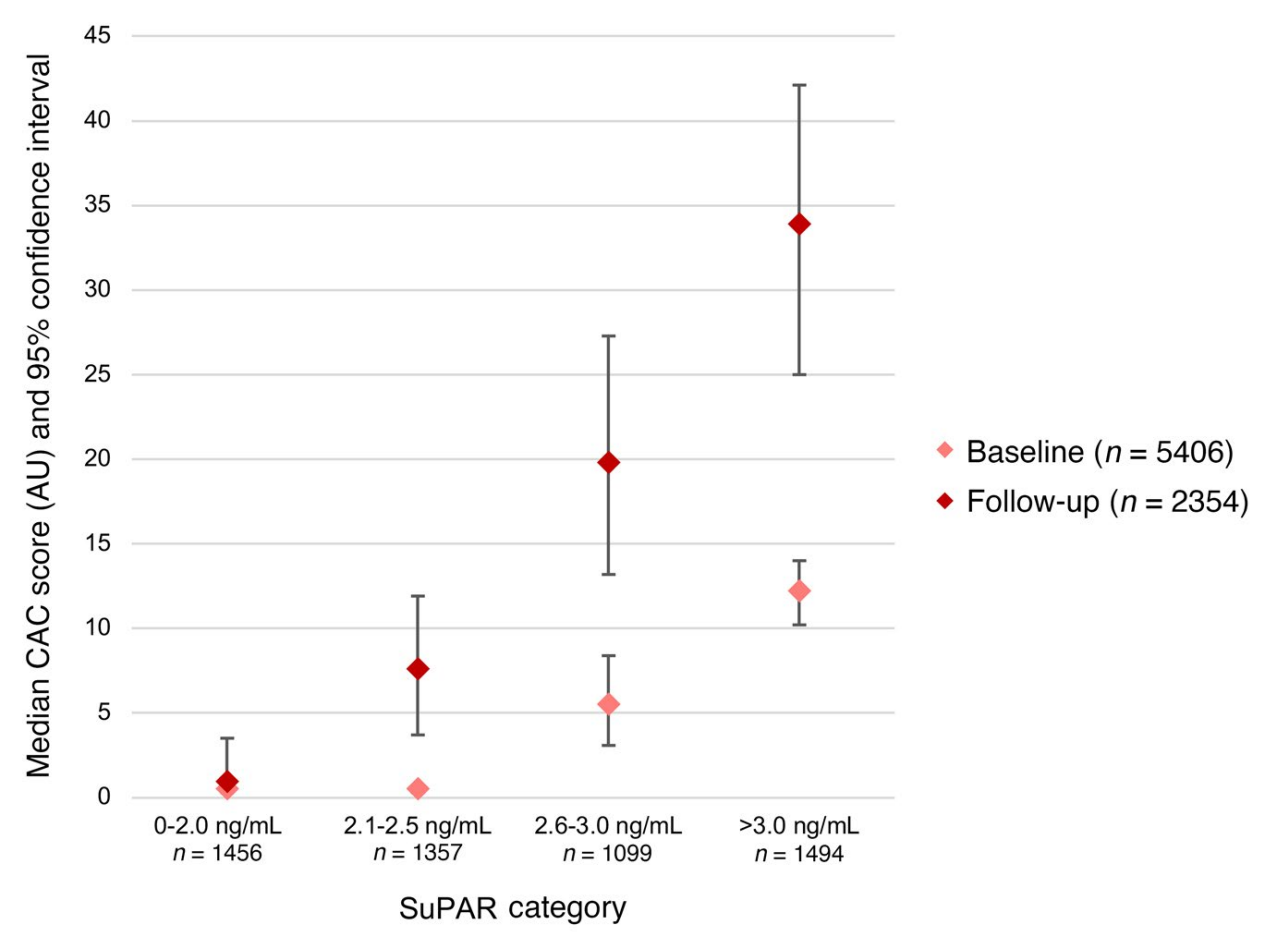
Median CAC score at baseline and follow-up by suPAR categories. Median CAC score (AU) based on Agatston scoring method at baseline and initial follow-up visits stratified by suPAR categories: 0–2.0 ng/mL, 2.0–2.5 ng/mL, 2.5–3.0 ng/mL, and >3.0 ng/mL. Error bars represent 95% CI.

The median time between baseline and initial follow-up measurement of CAC was 2.5 years. For all suPAR categories, the median CAC at follow-up was higher than baseline: participants with suPAR of less than 2.0 ng/ml had a 103% increase in CAC at follow-up compared with 229% for those with suPAR greater than 3.0 ng/ml (Figure 1 and Supplemental Figure 1). In multivariable analysis, higher baseline suPAR levels were associated with a greater increase in CAC scores over time (Supplemental Table 3), with a yearly increase in CAC score of 15.0 AU (95%CI 6.6–23.4) per 2-fold higher suPAR levels.

### suPAR and cardiovascular outcomes in the multi-ethnic study of atherosclerosis

**Figure 2:**
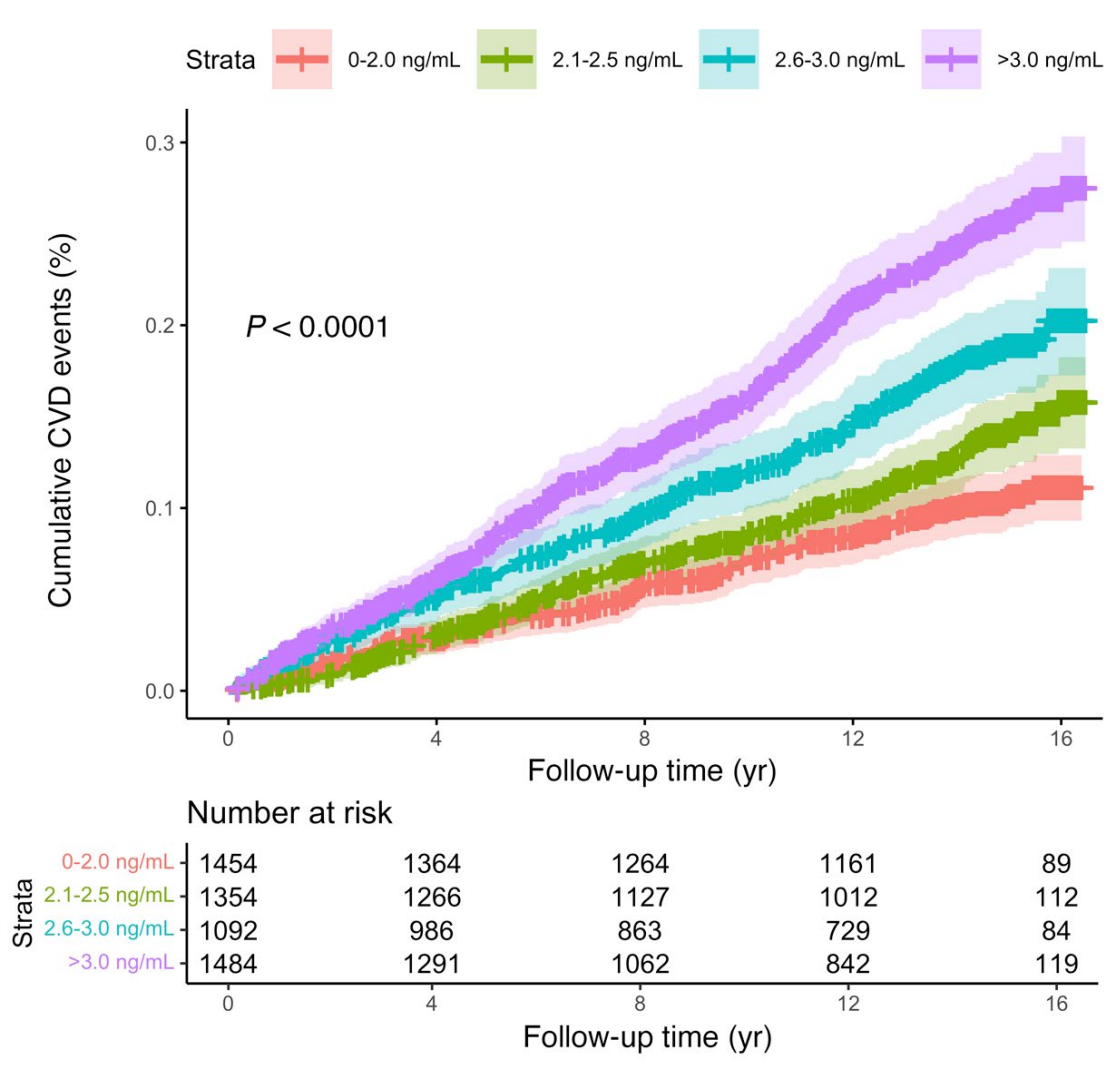
Cumulative incidence of any CVD event by suPAR categories. Unadjusted Kaplan-Meier curves for the cumulative incidence of CVD events stratified by suPAR categories: 0–2.0 ng/mL (red), 2.0–2.5 ng/mL (green), 2.5–3.0 ng/mL (blue), >3 ng/mL (purple). The difference in cumulative incidence curves between suPAR categories was tested using the log-rank test. A CVD event was defined as the composite of myocardial infarction, resuscitated cardiac arrest, angina, revascularization, stroke (excluding transient ischemic attack), and death due to CVD.

A total of 604 (11.2%) MESA participants developed incident CVD over a median (IQR) follow-up of 15 (10, 15) years, with 8.9 events per 1,000 person-years. Higher suPAR categories were associated with a higher incidence of CVD events in unadjusted and adjusted models (log-rank *P* < 0.001) (Figure 2 and Supplemental Figure 2). Participants with a baseline suPAR greater than 3.0 ng/mL had an incidence rate of 9.0 CVD events per 1,000 person-years (95% CI 7.7–10.7), whereas participants with a baseline suPAR between 0 and 2.0 ng/mL had an incidence rate of 2.8 events per 1,000 person-years (95%CI 2.2–3.7). In multivariable analysis, higher suPAR levels were associated with a higher risk of CVD events: 1.46-fold higher (95% CI 1.29–1.65) for each 2-fold higher suPAR level and 1.77-fold higher (95%CI 1.42–2.19) for participants with suPAR greater than 3.0 ng/mL compared with suPAR of less than 2.0 ng/mL (Supplemental Table 4). The association between suPAR and CVD events was not attenuated by adjusting for baseline estimated glomerular filtration rate (eGFR), longitudinal change in eGFR, baseline CAC, or baseline high-sensitivity troponin T and N-terminal–prohormone BNP (NT-proBNP) (Supplemental Figure 3 and Supplemental Tables 5 and 6) and did not differ according to the presence (CAC > 0) or absence of CAC (CAC = 0) at baseline (*P* interaction = 0.31) or baseline eGFR (*P* interaction = 0.98).

### GWAS meta-analysis of suPAR

We performed a multi-ancestry GWAS meta-analysis of suPAR levels (Supplemental Figure 4) on 16.6 million variants in 12,937 participants of 4 cohort studies with European (*n* = 9,869), African (*n* = 1,363), East Asian (*n* = 623), and Hispanic (*n* = 1,082) ancestries. Fifteen independent signals in 8 loci were associated with suPAR levels at a genome-wide significance level (*P* < 5 × 10^–8^) (Table 1 and Supplemental Figure 5). A meta-analysis limited to the European ancestry sample included 9.9 million variants and identified 12 independent signals in 8 loci at genome-wide significance (Supplemental Figure 5 and Supplemental Table 7).

The strongest signals in both GWAS analyses were in or near the genes encoding suPAR (*PLAUR*) and its canonical ligand uPA (*PLAU*). There were 6 independent signals at the *PLAUR* locus, and 2 of them included missense variants in the *PLAUR* gene (Supplemental Figure 6): rs2302524 (p.Lys220Arg in domain III of uPAR), with each minor C allele associated with a 0.21 SD increase in suPAR levels (*P* = 1 × 10^–35^); and rs4760 (p.Leu317Pro in the C-terminal portion of the proprotein uPAR form), with each minor G allele associated with a 0.11 SD increase in suPAR levels (*P* = 8 × 10^–9^). Four other putatively independent signals were tagged by top SNPs in the noncoding sequence of the *PLAUR* locus (Table 1). At the *PLAU* locus, the A allele of rs2633321 was associated with higher levels of suPAR (β = 0.10 SD, *P* = 6 × 10^–15^). Associations between suPAR levels and 12 of the 15 signals, including the 2 *PLAUR* missense variants, were replicated in the DBDS cohort (36) (*n* = 12,177) (Table 1 and Supplemental Table 7).

The regional association plots for all 8 loci from the European ancestry meta-analysis are illustrated in Supplemental Figure 7. The variance of suPAR levels explained by a weighted genetic risk score of all independent variants was 3%. Using sequential conditional analysis, 3 of the top 6 variants at the *PLAUR* locus (rs4760, rs2302524, and rs36229204) remained independent (Supplemental Figure 8). Bayesian fine mapping of the *PLAUR* locus resulted in 5 credible sets with both missense variants, rs4760 and rs2302524, capturing the top 2 of the 5 sets with very high posterior inclusion probabilities (Supplemental Table 8). The other 3 sets were captured by 3 sentinel variants, rs4251824, rs117564136, and rs400058.

### Impact of PLAUR missense variants on suPAR levels in vitro and in vivo

**Figure 3:**
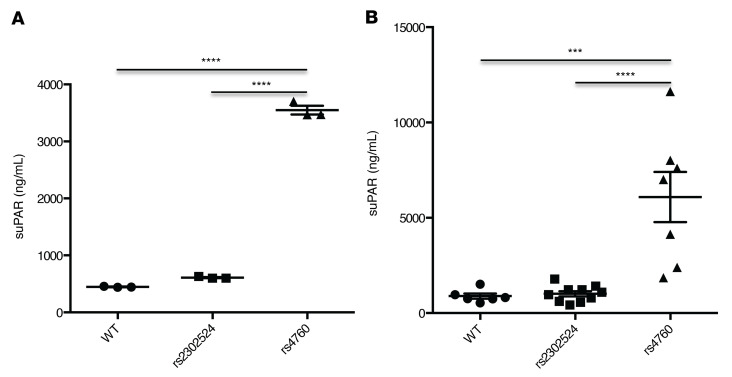
In vitro and in vivo expression of *PLAUR* missense variants and suPAR levels. Human suPAR levels in (**A**) supernatant of HEK cells 48 hours after transfection with rs4760 (*n* = 3) and rs2302524 (*n* = 3) *PLAUR* variants and in (**B**) C57BL/6J mice 24 hours after hydrodynamic tail-vein injection of plasmid DNA containing WT (*n* = 6) or the rs2302524 (*n* = 10) or rs4760 (*n* = 7) variant. ***P < 0.001; ***P < 0.0001, 1-way ANOVA.

To support the GWAS findings at the *PLAUR* locus, we assessed experimentally whether the *PLAUR* missense variants rs2302524 and rs4760 led to altered suPAR levels compared with the reference allele. We transfected HEK293 cells with plasmid DNA encoding either reference cDNA or the missense variants and measured suPAR levels in the cell media 48 hours later. The supernatant of cells transfected with the rs4760 variant had higher suPAR levels compared with reference, while we observed no increase in suPAR in the medium of cells transfected with rs2302524 (Figure 3A) compared with reference. We did not find significant differences in *PLAUR* gene expression or differing patterns of cellular distribution of uPAR on immunostaining among the reference and the rs4760 and rs2302524 variants in HEK293 cells (Supplemental Figure 9), suggesting that the increase in suPAR levels is caused by increased secretion and not mediated by an increase in expression or cellular redistribution.

Expression of rs4760 (p.317Pro) in vivo using mouse hydrodynamic tail-vein injection of plasmid DNA similarly demonstrated a significant increase in serum suPAR levels 24 hours after injection (Supplemental Figure 10), while rs2302524 had no significant difference compared with the reference sequence (Figure 3B). These findings confirm that the rs4760 variant, but not rs2302524, has a significant impact on suPAR levels, likely through increased secretion.

### Genetically predicted suPAR level and atherosclerotic disease in the UK Biobank

**Figure 4:**
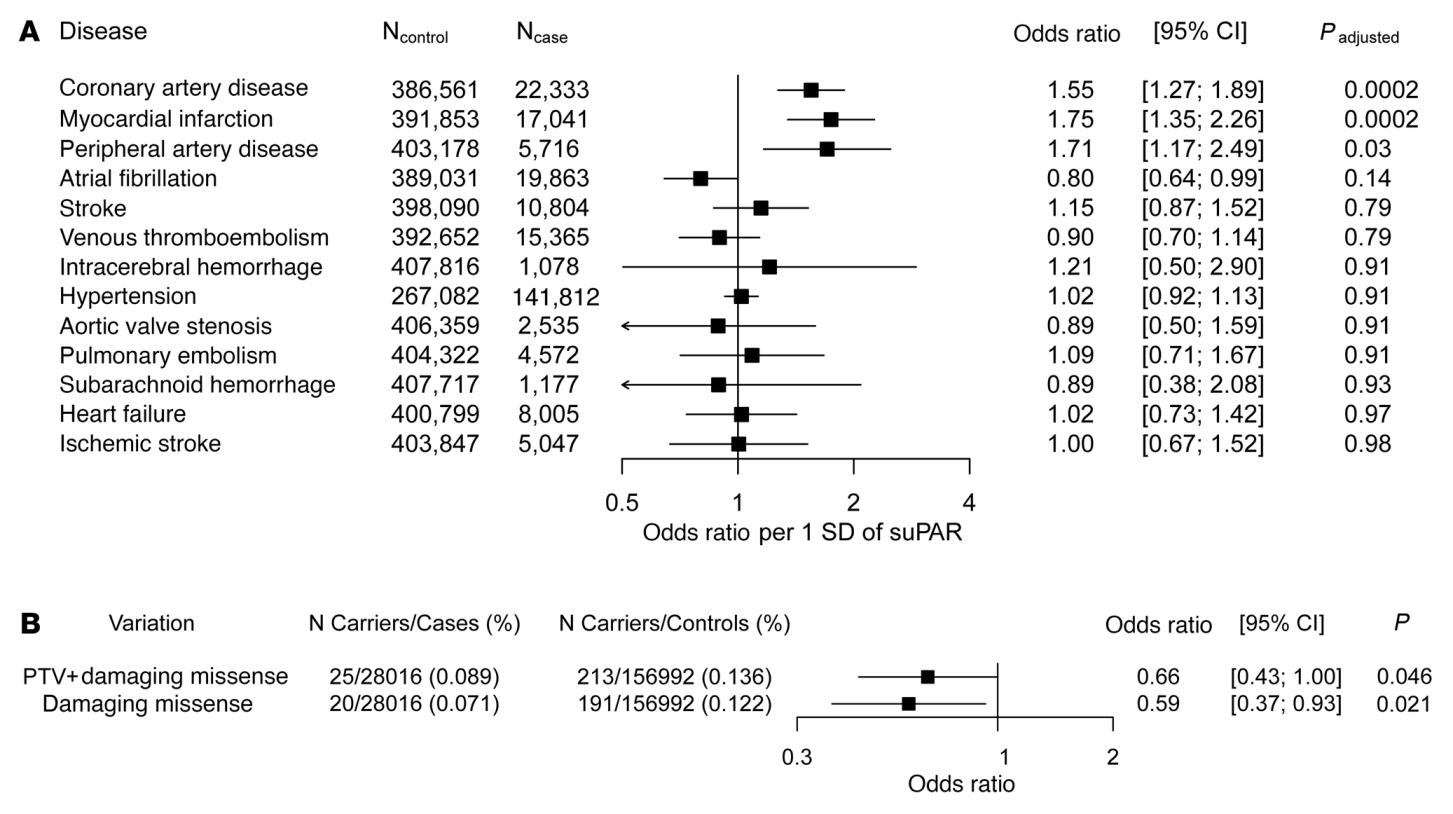
MR phenome-wide association of genetically predicted suPAR by rs4760 with CVD and rare damaging missense variants’ impact on the odds of ischemic heart disease. (**A**) Causal effect of suPAR on 13 CVDs by MR using missense variant rs4760 as instrument. Effect estimates are provided per 1 SD increase in suPAR levels. *P* values were adjusted using the false discovery rate method. (**B**) Rare variant gene collapsing analysis of the more than 280,000 exomes in the UK Biobank. Both rare protein truncating variants and rare damaging missense variants in the *PLAUR* gene were selected to study the impact of attenuated PLAUR function on coronary heart disease.

To assess whether suPAR levels are causally linked to CVD, we performed MR using the experimentally validated *PLAUR* rs4760 missense variant and the following cardiovascular phenotypes: aortic valve stenosis, atrial fibrillation, coronary artery disease, heart failure, hypertension, intracerebral hemorrhage, ischemic stroke, myocardial infarction, peripheral artery disease, pulmonary embolism, stroke, subarachnoid hemorrhage, and venous thromboembolism. We found that a genetically predicted 1 SD increment in suPAR was specifically associated with atherosclerotic phenotypes: 55% higher odds of coronary artery disease (*P* adjusted = 0.0002), 75% higher odds of myocardial infarction (*P* adjusted = 0.0002), and 71% higher odds of peripheral arterial disease (*P* adjusted = 0.03) after adjusting for multiple comparisons (Figure 4A). Associations with coronary artery disease and peripheral arterial disease were replicated in independent cohorts (CARDIoGRAM C4D, ref. 37; and the Million Veterans Program, ref. 38) (Supplemental Figure 11). We did not observe an association between rs2302524 variant suPAR and any of the cardiovascular phenotypes (Supplemental Figure 12).

We also found that higher suPAR levels predicted by rs4760 were associated with lower creatinine-derived glomerular filtration rate (1% decrease per 1 SD higher suPAR; *P* = 0.001) and increased risk for CKD (OR = 1.24 per 1 SD higher suPAR, *P* = 0.02) in the UK Biobank and CKDGen (39) consortium, supporting the hypothesis of suPAR being a common pathogenic factor between cardiovascular and kidney disease (Supplemental Figure 13).

To support the findings in MR, we performed a collapsing analysis of rare variants in *PLAUR*. Our hypothesis was that rare damaging variants in *PLAUR* would lead to reduced plasma suPAR levels and be associated with a reduced risk for the cardiovascular phenotypes implicated by MR. We examined the more than 280,000 exomes in the UK Biobank and found that individuals with rare nonbenign coding variants in *PLAUR* had a lower risk of ischemic heart disease. Aggregate burden of rare damaging coding variants was associated with 41% lower odds of ischemic heart disease (95% CI 7%–63%), suggesting that heterozygous loss of function of variants of *PLAUR* are protective against ischemic heart disease (Figure 4B).

### suPAR overexpression exacerbates atherosclerosis in a murine model

We then sought to determine whether experimentally raising levels of suPAR would exacerbate atherosclerosis. We induced atherosclerosis using proprotein convertase subtilisin/kexin-9–adeno-associated virus (*Pcsk9*-AAV) transfection in transgenic mice overexpressing full-length suPAR (*suPAR^Tg^*) and WT C57BL/6J mice. The *suPAR^Tg^* mice had total cholesterol levels similar to those of WT mice at baseline and after *D377Y-m Pcsk9* overexpression coupled with Western diet feeding for 10 weeks (Supplemental Figure 14). suPAR levels were significantly higher in *suPAR^Tg^* mice compared with WT at baseline (2.4 μg/mL versus 0.005 μg/mL, respectively) and at 10 weeks (25.2 μg/mL versus 0.01 μg/mL, respectively; Supplemental Figure 14).

**Figure 5:**
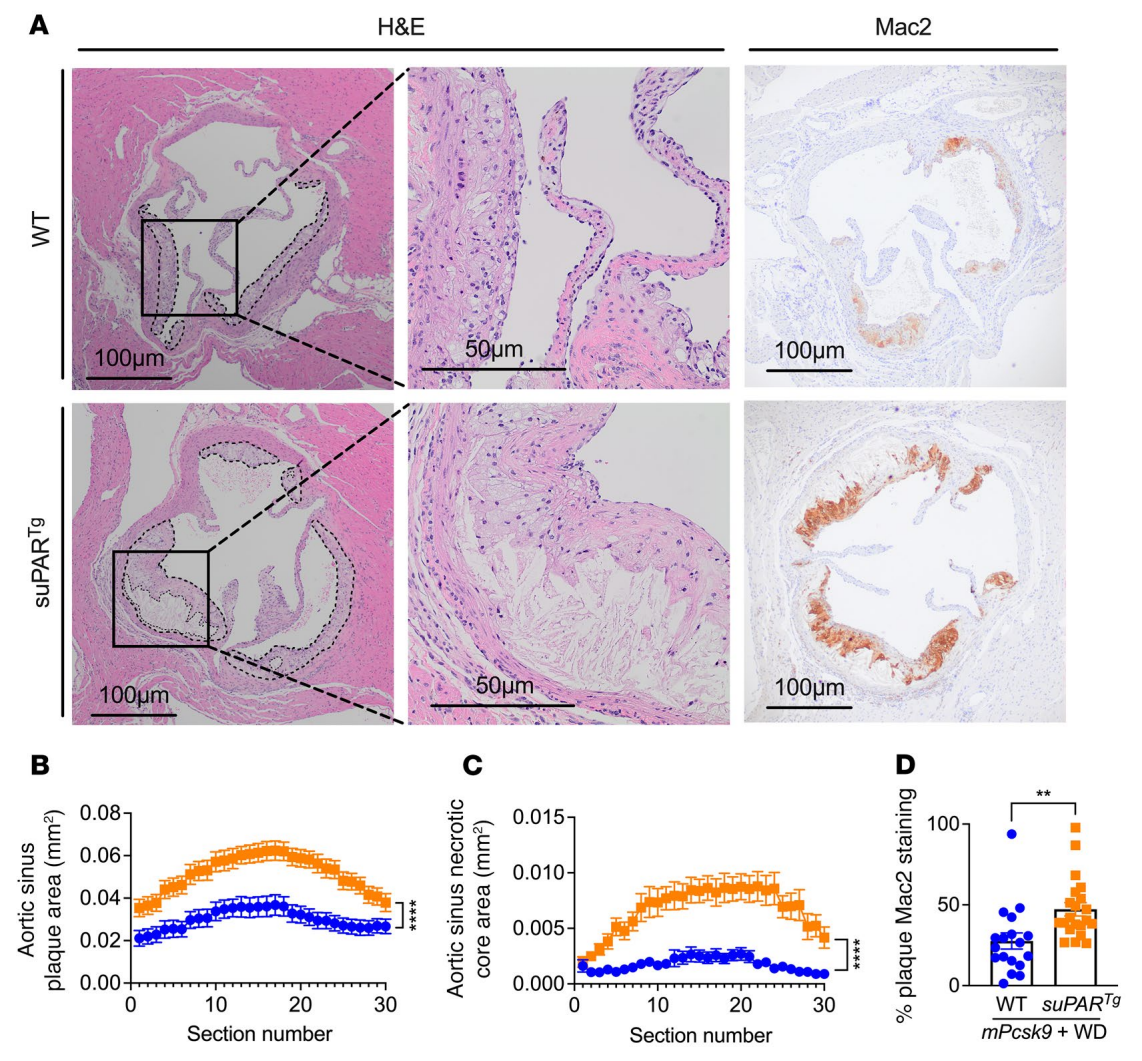
suPAR overexpression leads to increased atherosclerotic and necrotic plaques in a murine model of atherosclerosis. WT (*n* = 18) and *suPAR^Tg^* (*n* = 21) mice were maintained on a low-fat diet until 3 months of age and were then transfected with *Pcsk9*-AAV and fed a western diet (WD) for 10 weeks. At this point, aortic roots were obtained, paraffin embedded, and stained with H&E and Mac2 (galectin 3). (**A**) Cross sections of aortic roots from C57BL/6 WT and *suPAR^Tg^* mice show total lesion area, outlined in dashed lines, and necrotic core area, outlined in dotted lines. Higher magnification shows the presence of necrotic core. Mac2 monoclonal antibody stain shown on aortic sinus cross sections from WT and *suPAR^Tg^* mice. Scale bars: 100 μm; 50 μm. (**B** and **C**) Quantification of total lesion area and necrotic core area for all 30 sections. (**D**) Quantification of Mac2 staining as a percentage of total plaque area with necrotic area subtracted. Atherosclerotic plaque and necrotic core areas: *n* = 18 WT and *n* = 21 *suPAR^Tg^* groups. Tissue sections are 6 μm each with 6 μm blank section between for a total of one 360 μm through the aortic sinus. Each data point represents a biological replicate for **D**. **P < 0.01; ****P < 0.0001, 2-way ANOVA (**B** and **C**); Student’s *t* test (**D**).

All *suPAR^Tg^* (*n* = 21) mice developed larger plaques in the aortic root compared with the WT group (*n* = 18), with a mean plaque volume of 1.55 mm^3^ in the *suPAR^Tg^* and 0.90 mm^3^ in the WT group (Figure 5). Atherosclerotic plaques of the *suPAR^Tg^* mice had significantly increased necrotic core areas compared with WT mice, with a mean volume of 0.18 mm^3^ compared with 0.05 mm^3^, respectively. Observations were consistent when female and male mice were analyzed separately (Supplemental Figure 15). Furthermore, the atherosclerotic plaques of the *suPAR^Tg^* mice had a significantly higher percentage of macrophage-positive areas by Mac2 staining of 47.3% on average compared with 27.6% in the WT mice (Figure 5). suPAR was detectable in both WT and *suPAR^Tg^* atherosclerotic plaques, with increased deposition in *suPAR^Tg^* plaques compared with those in WT (Supplemental Figure 16).

### Elevated suPAR levels induce proatherogenic changes in monocyte profiles

**Figure 6:**
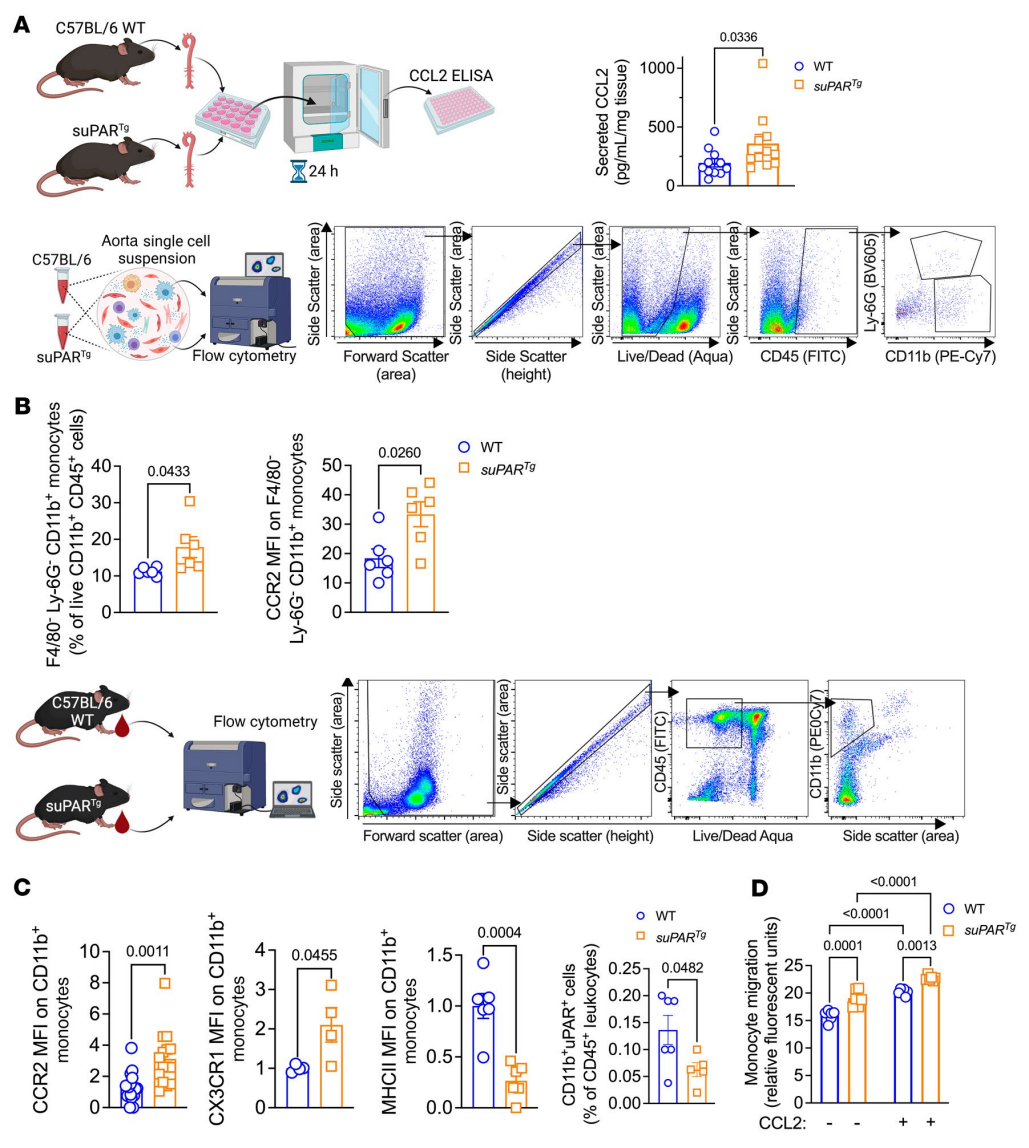
suPAR overexpression in mice leads to proatherosclerotic phenotype in circulating and aortic monocytes. Aortas and blood were harvested from disease-free C57BL/6 WT and suPAR overexpressing mice (*suPAR^Tg^* mice). (**A**) Aortas from WT (*n* = 11) and *suPAR^Tg^* (*n* = 11) mice were excised, cleaned of fat, and cultured for 24 hours. At this point, the conditioned culture medium was isolated and CCL2 level was assessed by ELISA. (**B**) Aortas from WT (*n* = 6) and *suPAR^Tg^* (*n* = 6) mice were isolated, cleaned of fat, digested, stained with fluorescently labeled antibodies, and analyzed by flow cytometry. Quantification of F4/80–Ly-6G–CD11b^+^ monocytes from WT and *suPAR^Tg^* mice as a percentage of live CD11b^+^CD45^+^ cells and median fluorescent intensity (MFI) of CCR2 expression from WT on F4/80–Ly-6G–CD11b^+^ monocytes. (**C**) Blood from WT and *suPAR^Tg^* mice was isolated and red blood cells were lysed, stained with fluorescently labeled antibodies, and analyzed by flow cytometry. MFI on live CD45^+^CD11b^+^ monocytes for expression of CCR2, MHCII, and CX3CR1, and percentage of uPAR^+^ cells of live CD45^+^CD11b^+^ cells. CCR2: *n* = 16 WT and *n* = 15 *suPAR^Tg^*, compared by Student’s *t* test. MHCII: *n* = 6 WT and *n* = 6 *suPAR^Tg^*. CX3CR1: *n* = 4 WT and *n* = 4 *suPAR^Tg^*. uPAR^+^ cells: *n* = 6 WT and *n* = 5 *suPAR^Tg^*. For MHCII, CX3CR1, and uPAR^+^ cells, Mann-Whitney U test was used. (**D**) Monocytes were isolated from spleens of WT and *suPAR^Tg^* mice and cultured in Transwell assays with either control cell culture media or cell culture media with CCL2 added. Quantification of fluorescent intensity of cellular dye was compared by 2-way ANOVA. *n* = 6 for each group. Each data point represents a biological replicate.

Given the urokinase receptor system’s known role in the regulation of innate immune system physiology, notably efferocytosis, we sought to assess whether suPAR overexpression altered the profile and function of monocytes and macrophages. We first examined aortas isolated from WT and *suPAR^Tg^* mice that did not undergo *Pcsk9*-AAV transfection to avoid the confounding effects of atherosclerosis and hyperlipidemia. We found that nonatherosclerotic *suPAR^Tg^* aortas secreted significantly higher levels of C-C motif chemokine ligand 2 (CCL2), one of the primary monocyte chemoattractants implicated in atherosclerosis, compared with WT aortas (Figure 6A) (40). Flow cytometry of aortic cell suspensions revealed a 2-fold higher count of monocytes in *suPAR^Tg^* aortas compared with WT (Figure 6B). The *suPAR^Tg^* monocytes isolated from aortas exhibited higher expression of C-C chemokine receptor type 2 (CCR2), the receptor for CCL2, compared with WT monocytes (Figure 6B). Circulating monocytes and bone marrow–derived macrophages exhibited a similarly proinflammatory phenotype, with higher expression of CCR2 and lower expression of major histocompatibility complex class 2 (MHCII) and membrane-bound uPAR (Figure 6C and Supplemental Figure 17). Circulating monocytes from *suPAR^Tg^* mice also exhibited increased expression of C-X3-C motif chemokine receptor 1 (CX3CR1), another chemokine receptor that has been implicated in atherosclerosis (41), compared with WT (Figure 6C).

We next assessed whether monocyte chemotaxis in *suPAR^Tg^* is altered as measured by migratory potential using a Transwell assay. Significantly more *suPAR^Tg^* monocytes migrated through the Transwell membrane compared with WT monocytes in response to both basal cell culture media and cell culture media with added recombinant CCL2 (Figure 6D). Overall, these data indicate that suPAR acts on monocytes and myeloid cells in general to render these cells more atherogenic (40–42).

## Discussion

We report epidemiologic, genetic, and experimental evidence of a causal role for suPAR in atherosclerosis. In a multi-ethnic cohort of over 5,000 participants without known CVD, we found high suPAR levels to be strongly associated with incident CVD and accelerated atherosclerosis as measured by serial CAC scores independently of decline in kidney function and established risk factors. In genetic analyses, we identified 2 independent common missense variants in *PLAUR* associated with higher plasma suPAR levels. One variant (rs4760, p.Leu317Pro) was confirmed experimentally in vitro and in vivo to lead to higher suPAR levels. Using that variant as an instrument in MR, we found that increased suPAR levels were causally linked to atherosclerotic phenotypes in the UK Biobank, notably coronary artery disease, myocardial infarction, and peripheral arterial disease in addition to kidney disease. Conversely, rare, damaging variants of *PLAUR* were associated with lower risk of ischemic disease. Experimentally, overexpression of suPAR in a murine model of atherosclerosis using *Pcsk9*-AAV led to a 2-fold increase in atherosclerotic plaque size with large necrotic cores and macrophage infiltration in *suPAR^Tg^* mice compared with WT mice.

Monocytes from *suPAR^Tg^* mice exhibited a proatherogenic profile and altered function even prior to the induction of atherosclerosis. Overall, chronically elevated suPAR levels appear to promote atherosclerosis at least partially through priming the immune system to a dysregulated response. These findings dovetail extensive experimental and clinical data on suPAR’s role in kidney disease and place high suPAR levels as a shared risk factor and potential therapeutic target for CVD and CKD.

Systemic inflammation is recognized as a key process common to CVD and CKD, with suPAR levels traditionally perceived as biomarkers of chronic inflammation related to activation of the innate immune system (7, 43, 44). suPAR levels are induced by shared risk factors for CKD and CVD, such as smoking, hypertension, and diabetes mellitus (14, 19, 22), associated with coronary and peripheral atherosclerotic disease (23, 25, 28, 45–47) and are predictive of incident kidney disease and CVD outcomes across age, sex, race, and clinical settings, independently of the aforementioned risk factors (11, 12, 14–16, 18, 19, 24, 26–34). To determine whether high suPAR levels precede CVD, we leveraged MESA — a cohort in which clinical CVD was an exclusion criterion at enrollment – and found that high suPAR levels at baseline predicted accelerated atherosclerosis as measured by CAC and incident CVD events even in participants with CAC equal to 0 and normal kidney function. Other biomarkers of inflammation have not exhibited a similar relationship with CAC (48, 49), which has prompted us to further explore suPAR’s singular role in atherosclerosis — now supported by our genetic and experimental analyses.

GWAS have revealed connections between common genetic variants and the risk for complex disease traits and quantitative traits such as plasma protein concentrations (50). These genetic variants, which are inherited independently of other disease risk modifiers, can be used in MR studies to determine whether a specific protein plays a causal role in a complex disease (51). We report a single instrument MR (rs4760) that supports a causal role for suPAR in the pathogenesis of atherosclerosis. MR studies rely on the assumption that the instruments (SNPs) used in the analysis are a genetic proxy for only one action, namely, altered levels of the protein being tested. This assumption is very likely to be valid with rs4760. This SNP is located only in the proprotein form of uPAR and results in higher levels of full-length (DI-DII-DIII) reference sequence suPAR in circulation. Thus, even though rs4760 may be associated with other traits, these are likely mediated by altered suPAR levels and not through pleiotropic effects on genes other than *PLAUR*. The heterogeneity that we noted between rs2302524 and rs4760 may relate to the functional consequences of the missense variant on suPAR in the pathogenesis of atherosclerosis. Although the rs2302524 variant was the top signal in GWAS, it did not lead to an increase in levels when expressed experimentally and was not found to be linked to CVD phenotypes in a previous study (52). The resulting amino acid change encoded by rs2302524 (p.Lys220Arg) is located in the DIII domain of suPAR and is associated with levels of variant suPAR in humans. Query of the Genotype-Tissue Expression (GTEx) database (https://gtexportal.org/home/gene/PLAUR) for *PLAUR* expression in human tissues revealed that the rs2302524 C allele is associated with lower *PLAUR* gene expression and that this association is the opposite of that observed with plasma suPAR levels. According to GTex, the rs2302524 is a splicing quantitative trait locus associated with higher levels of alternative splicing of *PLAUR* transcripts, resulting in the expression of different suPAR isoforms. The lack of colocalization of this variant and the cardiovascular phenotypes could be due to impaired function of the p.Lys220Arg suPAR secondary to the missense variant or due to altered circulating isoforms of suPAR. Conversely, the rs4760 variant is associated with increased plasma suPAR levels without altering the structure of the circulating protein, as the p.Leu317Pro variant is located only in a proprotein form of uPAR, suggesting that full-length (DI-DII-DIII) reference suPAR is the pathogenic form.

To confirm whether high levels of full-length suPAR accelerate atherosclerosis, we used a murine *Pcsk9*-AAV model of induced atherosclerosis, which allows for the study of immunometabolic processes without the confounding effects of germline alterations seen with the apoE knockout and LDL receptor knockout models (53). We found that overexpression of suPAR led to a 2-fold increase in total atherosclerotic plaque size, a 3.5-fold increase in necrotic core size, and a 2-fold increase in lesional macrophage infiltration in *suPAR^Tg^* compared with WT mice, without differences in cholesterol levels. Given the urokinase receptor system’s known role in modulation of immune cell motility and efferocytosis (3–9, 54, 55), we sought to determine whether chronically elevated suPAR levels affect monocyte profile and function in *suPAR^Tg^* mice that did not undergo induction of atherosclerosis. We found that nonatherosclerotic aortas of *suPAR^Tg^* mice excreted substantially higher CCL2 levels and contained more monocytes compared with aortas from WT mice.

Circulating monocytes of *suPAR^Tg^* mice exhibited higher expression of CCR2 and CX3CR1. The CCL2/CCR2 and CX3CR1 pathways have important roles in orchestrating monocyte recruitment into the vessel wall by chemotaxis, which we have found to be enhanced in *suPAR^Tg^* monocytes compared with WT (40, 41, 56). Moreover, monocytes from *suPAR^Tg^* also had reduced uPAR and MHCII expression compared with monocytes from WT mice. A decrease in MHCII expression and subsequent ability to present antigens has recently been linked to atherosclerosis through impairment of regulatory T cell activation (42), while a reduction in cell membrane–bound uPAR expression inhibits the “don’t eat me” signal, resulting in enhanced phagocytosis and efferocytosis (54, 57, 58). Conversely, macrophages derived from uPAR-knockout mice have impaired phagocytic ability (50). Overall, these data suggest that chronically elevated suPAR levels prime myeloid cells to be more atherogenic, leading to accelerated atherosclerosis in the setting of additional injurious stimuli (such as hyperlipidemia in this case). suPAR’s role in atherosclerosis may also be related to its binding of integrins (59). Integrins, notably α_v_β_3_, are crucial in initiation of atherosclerosis in endothelial cells and promote inflammation through the NF-κB pathway (59–61). Activation of integrins can also facilitate immune cell homing to the aorta and vascular remodeling (62). Future research will determine whether suPAR drives atherosclerosis through other mechanisms.

Overall, the greatest strength of this study is the multipronged approach to identifying a role for suPAR in atherosclerosis, leveraging epidemiologic and genetic analysis of large, well-characterized cohorts, and using an experimental murine model of atherosclerosis that does not involve germline alterations. We acknowledge certain limitations. The use of CAC as a surrogate for atherosclerosis may have led to an underestimation of the strength of its association with suPAR in MESA, given CAC scoring does not identify noncalcified plaque or coronary stenoses. Our genetic findings are disparate from a recent study that inferred genetic determinants of suPAR levels measured using proteomics platforms (63). The correlation between suPAR levels measured with proteomics platforms and immunoassays is, however, poor (*r* = 0.2–0.5), and their associations with outcomes vary greatly (64–68), explaining why our results differed from those obtained using proteomics platforms. Our GWAS analysis encompasses over 24,000 individuals in whom suPAR levels were measured using the suPARnostic (ViroGates) immunoassay used in the seminal studies on suPAR, kidney disease, and CVD outcomes (14, 18, 28, 34, 67). While our analysis included participants of mostly European ethnicity, a smaller GWAS in Black individuals also identified rs4760 as a determinant of suPAR levels measured using immunoassay (69). Finally, our experimental approach relies on the use of murine models of atherosclerosis, which cannot recapitulate all the features of the human disease, with major differences in lipoprotein metabolism and bile acid absorption (70). Nevertheless, these models are commonly used and have provided valuable insights into the pathophysiology of atherosclerosis (53).

Our findings may have important implications. suPAR’s role in modulating the inflammatory profile and function of myeloid cells likely extends beyond atherosclerosis and may represent a common mechanism underlying suPAR’s role as a predisposing factor in other chronic diseases, such as kidney, rheumatologic, and inflammatory bowel diseases. Targeting inflammation as a strategy for decreasing the risk of CVD has been shown to be viable in recent trials using monoclonal antibodies to IL-1β and IL-6 (40, 71, 72). suPAR has been targeted successfully in experimental models; bone marrow ablation (10), monoclonal antibodies directed to suPAR (8, 18), or small molecule inhibitors of suPAR can prevent or reverse kidney injury (21). In patients with focal segmental glomerulosclerosis, plasmapheresis reduces suPAR levels, decreases β_3_ integrin activity, and stabilizes the disease (73–75). The aggregate of epidemiologic, genetic, and experimental evidence we provide and the advent of anti-suPAR therapies strongly support exploring suPAR as a target for the prevention and treatment of CVD.

## Methods

### suPAR, CAC, and incident cardiovascular events

#### Study cohort.

MESA is a multicenter observational cohort designed to identify risk factors for the incidence and progression of CVD. A detailed description of the study design and methods has been published previously (76). In summary, 6,814 (3,601 women; 3,213 men) participants aged 45 to 84 years who identified as either White, Black, Hispanic, or Chinese were enrolled between 2000 and 2002 at 6 participating communities across the US. Participants were eligible if they were free of clinical CVD at enrollment. For the present study, we included all participants who provided serum samples for suPAR biomarker measurements at enrollment (*n* = 5,406).

#### Measurement of CAC.

A detailed description of the methodology for the acquisition and interpretation of CAC scores in MESA has been published previously (77). Briefly, CT scanning of the chest was performed using either electron-beam CT (Chicago, Los Angeles, and New York field centers) or using a multidetector CT system (Baltimore, Maryland, USA; Forsyth County, Georgia, USA; and St. Paul, Minnesota, USA, field centers). CAC scores were calculated using the Agatston score and adjusted with a standard calcium phantom that was scanned with the participant (78). The mean Agatston score (AU) was used in all analyses. Interobserver and intraobserver agreement were high (*k* statistic = 0.90 and 0.93, respectively). CAC scores were measured at baseline (exam 1: July 2002–August 2002) with initial follow-up measurements performed on half of the cohort at exam 2 (September 2002–January 2004) and the other half at exam 3 (March 2004–July 2005). A quarter of participants were selected for CAC measurement at exam 4 (September 2005–May 2007).

#### Measurement of suPAR.

suPAR was measured using a commercially available ELISA (suPARnostic, ViroGates) in serum samples. The lower limit of detection of the assay is 100 pg/mL; however, all measurements were above the lower limit of detection. The interassay coefficient of variation determined using blinded replicate samples from participants ranged from 8% to 11%, depending on the cohort. suPAR levels are stable in stored serum samples, with levels reproducible in samples stored for over 5 years at –80°C (79).

#### suPAR and CAC.

Clinical characteristics for the cohort are reported stratified by suPAR categories (0–2.0 ng/mL, 2.0–2.5 ng/mL, 2.5–3.0 ng/mL, and > 3.0 ng/mL). We examined the correlation between suPAR and CAC scores at baseline using Spearman’s rank. To determine whether suPAR levels (log-transformed base 2) were independently associated with CAC at baseline, we used linear regression with CAC as the dependent variable adjusted for CVD risk factors including age, sex, race, BMI, history of smoking, eGFR using the Chronic Kidney Disease Epidemiology Collaboration equation (80), LDL levels, HDL levels, C-reactive protein, hypertension (use of antihypertensives or systolic blood pressure ≥140/90 at enrollment), and diabetes mellitus. We then visualized the median CAC scores at baseline and initial follow-up stratified by suPAR categories using bar graphs. Additionally, we examined the adjusted difference in CAC scores between baseline and initial follow-up by calculating the mean predicted change in CAC score for each suPAR category accounting for age, sex, race, BMI, history of smoking, eGFR, LDL levels, HDL levels, C-reactive protein, and diabetes mellitus.

To determine whether suPAR levels at baseline were associated with an increase in CAC over time, we generated generalized estimating equations modeling with CAC as a continuous and longitudinal variable using all CAC scores measured after baseline and examined the interaction term suPAR × follow-up time. The model was adjusted for the aforementioned variables in addition to baseline CAC.

#### suPAR and cardiovascular events.

We then assessed whether suPAR levels were predictive of CVD events. A CVD event was defined in MESA as the composite of myocardial infarction, resuscitated cardiac arrest, angina, revascularization, stroke (excluding transient ischemic attack), or death due to CVD (76, 77). We used stepwise multivariable-adjusted Cox’s proportional hazards modeling to assess the contribution of relevant factors such as eGFR and CAC to the association between suPAR and CVD events. Model 0 (suPAR alone) was unadjusted; model 1 was adjusted for age, sex, race, BMI, history of smoking, LDL, HDL, C-reactive protein, hypertension, and diabetes mellitus; model 2 included all variables in model 1 in addition to baseline eGFR; and model 3 included the variables in model 2 with the addition of baseline CAC. We explored eGFR as a time-varying covariate in a separate model including the covariates from model 3. In MESA, eGFR was measured at baseline and at exam 5 (April 2010–February 2012). suPAR was modeled as a continuous (log-transformed base 2) and categorical variable (0–2.0 ng/mL, 2.0–2.5 ng/mL, 2.5–3.0 ng/mL, and > 3.0 ng/mL) in all models. Additionally, we conducted a sensitivity analysis, further adjusting for baseline high-sensitivity troponin T and NT-proBNP in addition to the variables in model 2. Follow-up time was up to the first CVD event, death, last contact with the research team, or end of study period. Unadjusted and adjusted Kaplan-Meier cumulative incidence curves for CVD events were generated. Adjusted Kaplan-Meier curves were calculated using inverse probability weighting for suPAR categories with propensity scores estimated using generalized boost models adjusted for age, sex, race, BMI, history of smoking, eGFR, LDL levels, HDL levels, C-reactive protein, and diabetes mellitus (81). A complete case analysis was performed. A 2-sided *P* value of less than 0.05 was used to determine statistical significance. Analyses were performed using R, version 4.1.0, (R Foundation for Statistical Computing).

### Genetic determinants of suPAR and the link to atherosclerosis

We measured plasma suPAR levels using immunoassay (suPARnostic,ViroGates) in 4 different cohorts: the Trinity Student Study (TSS) (82), the Genes and Blood-Clotting cohort (GABC) (83), MESA, and the Malmo Diet and Cancer Study (MDCS), totaling 12,937 participants (84). We performed GWAS and meta-analysis to identify genetic determinants of suPAR levels and replicated our findings in 12,177 healthy participants of the DBDS where suPAR levels were measured using the same immunoassay. The top 2 significantly associated missense variants of *PLAUR* were then expressed in human embryonic kidney cells (HEKs) (CRL-3216; ATCC) and in C57BL/6J mice (000664; Jackson Laboratory) to determine which variants led to significant increases in suPAR levels. We then leveraged the UK Biobank to perform MR and assess for a causal link between genetically determined suPAR levels and CVD (*n* = 408,894) (85).

#### GWAS cohorts and analysis.

The TSS is a cohort of 2,179 unrelated healthy and ethnically Irish individuals between 21 and 24 years old (59% women, 41% men, all European ancestry) (82). The GABC cohort comprises 931 young and healthy students between 14 and 35 years of age (63% women, 37% men, all European ancestry) (83). The MESA cohort included 5,092 unrelated participants aged 45 to 84 years (53% women, 47% men, 38% European ancestry, 28% African American, 22% Hispanic American, and 11% Chinese American) free from CVD (76). The MDCS is a Swedish population-based cohort that included 4,735 randomly selected unrelated participants between 44 and 73 years of age (59% women, 41% men, all European ancestry) (84). Finally, the DBDS genomic cohort comprises a subset of 12,177 healthy blood donors aged 18 to 66 years (47% women, 53% men, all European ancestry) (36).

Quality control measures were performed to exclude low-quality samples and low-quality variants within each study prior to imputation to reference genomes. In general, samples were excluded if they showed discordance between genetically inferred and reported sex, low call rate, and duplications. Variants were excluded if they deviated from the Hardy-Weinberg equilibrium.

Imputation was done to predict nongenotyped variants. The TSS, GABC, and MESA were imputed using TOPMed Freeze 5b (GRCh 38). The MDCS was imputed using the Haplotype Reference Consortium reference panel (GRCh 37) (86). The build was liftover to GRCh 38 using CrossMap (87). The DBDS was imputed using 1 KG phase 3, HapMap, and a data set consisting of more than 6,000 Danish whole-genome sequences.

#### GWAS analyses.

GWAS analyses were performed with natural log suPAR levels adjusted for age, sex, and the first 10 principal components of ancestry followed by inverse-normal transformation within each study and ancestry combination using array data imputed to reference genomes. Single-variant association analyses were performed using linear regression in PLINK, version 2.0 (88), within each study-ancestry combination. For GABC, linear mixed models incorporating a kinship matrix were performed using RVTESTS (89). Overall, our analyses resulted in genome-wide summary data from European ancestry data sets from MDC (*n* = 4,735), TSS (*n* = 2,179), MESA (*n* = 2,024), and GABC (*n* = 931), and African (*n* = 1,363), East Asian (*n* = 623) and Hispanic (*n* = 1,082) populations from MESA. We performed quality control measures on each of the summary association data sets prior to meta-analysis (90, 91). Within each data set, we filtered out variants with minor allele count of less than 20, Hardy-Weinberg equilibrium *P* value of less than 5 × 10^–6^, low imputation quality (INFO < 0.6), multiallelic variants, and palindromic variants (A/T or C/G) with minor allele frequency above 0.4.

#### Meta-analysis.

We performed multi-ancestry and European ancestry–specific inverse-variance weighted fixed effects meta-analyses using METAL software (90). We generated quantile-quantile plots to assess for genomic control and structure within our data (Supplemental Figure 18). To identify leading and independent variants from each meta-analysis, we performed pruning and thresholding using the “clump” flag in PLINK. PLINK implements an iterative multistep process in which variants are sorted by their *P* values and those in linkage disequilibrium are removed (*r^2^* < 0.05 and within 250 kilobases from the lead variant). The process is repeated until the genome-wide significance threshold of 5 × 10^–8^ is reached. The *PLAUR* locus was further finemapped using the SuSie Iterative Bayesian Stepwise Selection procedure (92). Top variants were defined as those with a *P* value of less than 5 × 10^–8^ and were independent of each other. We then investigated the identified variants in the DBDS cohort. Functional annotations for top variants were obtained from the Ensemble Variant Effect Predictor (91).

#### In vitro and in vivo expression of PLAUR missense variants.

We generated the *PLAUR* variants rs2302524 and rs4760 (Supplemental Figure 19) using the GeneArt site-directed mutagenesis system (Thermo Scientific) and WT *PLAUR* (NCBI’s RefSeqGene LRG_637 and RefSeq NG_032898.1) cloned into a pCMV6-entry vector (Origene).

Equal amounts (12 μg) of plasmid DNA encoding vector control, human reference, or the *PLAUR* missense variants were transfected into HEK293T cells (CRL-3216; ATCC) using the FuGENE 6 transfection reagent (E2691; Promega). The conditioned media and cells from each plate were harvested 48 hours after transfection for performing the following: (a) assess uPAR distribution with immunofluorescence staining of cells using monoclonal uPAR antibody to uPAR domain 2 (NBP2-62800, 1:400, Novusbio) and membrane marker P-cadherin (ab16505; 1:100, Abcam); (b) quantification of gene expression using real-time quantitative PCR testing; and (c) suPAR measurement in the supernatant using the Human uPAR Quantikine ELISA Kit (DUP00; R&D Systems).

We performed hydrodynamic tail-vein injection of plasmid DNA encoding reference human *PLAUR* (*n* = 5), *PLAUR* variant rs2302524 (*n* = 9), and *PLAUR* variant rs4760 (*n* = 7) in 8-week-old C57BL/6J female mice and measured serum suPAR levels 24 hours after injection using the Human uPAR Quantikine ELISA Kit.

#### MR analysis.

We leveraged the UK Biobank for MR analysis in 408,894 participants of European ancestry (UK Biobank resource, application number 59206) (93). Details of measures for variant and sample quality control have been previously reported (94). We used rs4760, the *PLAUR* missense variant confirmed to alter suPAR levels in both in vitro and in vivo models, as an instrument for MR analyses of 13 cardiovascular phenotypes from the UK Biobank (Supplemental Table 9). Significant associations were replicated using publicly available summary GWAS data from the CARDIoGRAM C4D consortium for coronary artery disease (60,801 cases and 123,504 controls) and the Million Veterans Program for peripheral arterial disease (31,307 cases and 211,753 controls) (37, 38). Wald ratios were used to derive the odds ratio per 1 SD increments in suPAR levels instrumented by rs4760. Similar analyses were performed using the rs2302524 missense variant as an instrument. Finally, we obtained summary-level data from the CKDGen consortium to perform MR and assessed for a causal link between genetically determined suPAR levels by rs4760 and (a) kidney function as measured by creatinine-derived eGFR (*n* = 567,460) (39) and (b) CKD (41,395 cases, 439,303 controls), defined as an eGFR of less than 60 ml/min/1.73 m^2^ (95). The MR was then replicated in the UK Biobank (eGFR, *n* = 387,937; CKD, 8,031 cases and 400,863 controls) (85). MR analyses were performed using the TwoSampleMR package in R, version 4.0.

To assess whether rare coding variations with damaging consequences on the suPAR protein are associated with ischemic heart disease, we performed a lookup in a previously published exome-sequenced analysis of more than 280,000 UK Biobank participants (http://azphewas.com/). Both rare protein truncating variants and rare damaging missense variants in the *PLAUR* gene were selected for studying the impact of attenuated PLAUR function on coronary heart disease. In brief, protein-truncating variants are defined as variants that are predicted to truncate a protein and with a maximum minor allele frequency of 0.001. Rare damaging missense variants were defined as those with a REVEL score of 0.25 or more and a maximum minor allele frequency of 0.0005 (96).

### suPAR overexpression in a Pcsk9-AAV murine model of atherosclerosis

A total of 39 mice, 12 to 16 weeks of age, including *n* = 18 C57BL/6J WT mice (000664, Jackson Laboratory), of which 7 were female, and *n* = 21 *suPAR^Tg^* mice, of which 4 were female, overexpressing the soluble form of mouse full-length suPAR (corresponding to NP_035243, DI-DII-DIII without GPI anchor) in adipose tissue using the adipocyte fatty acid binding protein (AP2) promoter on C57BL/6 background, were used (10). All mice were maintained on a 12-hour light/12-hour dark cycle with free access to food and water.

To induce hypercholesterolemia, we administered an i.p. injection of recombinant AAV8–D377Y–murine *Pcsk9* (5 × 10^6^ viral genomes/kg body weight), which was previously described (97). After 1 week, the diet was switched to a Western diet (42% calories from fat, Teklad, catalog 88137) for 10 weeks and all 39 mice completed the study.

#### Cholesterol and suPAR measurements.

Plasma was collected via tail-vein puncture in heparin-coated tubes. Fasting cholesterol levels were measured by colorimetric assay (STA-384; Cell Biolabs). Plasma levels of suPAR were measured using R&D DuoSet ELISA antibodies and Ancillary Reagent Kit 2 for development of a sandwich ELISA (DY531, R&D Systems). The ELISA has a detection range of 78 to 5000 pg/mL.

#### Atherosclerotic lesion analysis, histology, and immune histochemistry.

Mice were euthanized via carbon dioxide overdose. Blood was harvested by right ventricular puncture and the vasculature perfused with ice-cold PBS. The heart and brachiocephalic artery (BCA) were harvested from all 39 mice, placed in 4% paraformaldehyde, and embedded in paraffin. Sixty sections (6 μm each) were cut through the aortic root as the primary site of atherosclerosis, and 30 sections (6 μm each) were cut through the BCA as a secondary anatomic site from each mouse, as recommended (98). For morphometric analysis, 30 sections from the aortic root and 15 sections from the BCA were stained with H&E and assessed for total lesion size and necrotic core size (acellular lesion area) as previously described (99), for a total coverage of 360 μm of the aortic root. Paraffin-embedded sections of the aortic sinus were deparaffinized and rehydrated. After blocking, sections (6 μm each) were incubated at room temperature for 2 hours with Mac2 (sc-81728; Santa Cruz Biotechnology Inc.). Mac2 slides were counterstained with hematoxylin and coverslipped. Images were captured with an Olympus LC30 camera mounted on an Olympus CX41 microscope. For the Mac2^+^ area, all images were obtained with the same light source at the same time. The Mac2^+^ area was determined using the threshold function in ImageJ (NIH) and normalized to total nonnecrotic lesion area. Results were reported as percentage of lesion area. Sectioning and staining were performed by the In Vivo Animal Core Laboratory technicians at the Unit for Laboratory Animal Medicine, University of Michigan. Technicians in this laboratory were blinded to experimental identity. Atherosclerotic plaque size was calculated using ImageJ software and graphed by section number.

#### Ex vivo aorta culture and CCL2 measurement.

Thoracic aortas from C57BL/6J WT mice and *suPAR^Tg^* mice were excised and cultured in DMEM plus 10% fetal bovine serum with 1% penicillin-streptomycin solution (P4333; Sigma-Aldrich) at 37°C for 24 hours. Conditioned culture supernatants were collected and stored at –80°C. CCL2 levels in conditioned media were measured using ELISA (88-7391-22, Thermo Fisher Scientific).

####  Flow cytometry of aortic cell suspension and circulating cells.

Approximately 50 to 100 μl whole blood was harvested via tail vein, and the red blood cells were lysed using red blood cell lysis buffer (420302, BioLegend). Cells were then centrifuged for 5 minutes at 400*g*, and the supernatant was poured off.

Thoracic aortas were harvested into 1× PBS on ice, then minced and digested in 1× HBSS containing 450 U/mL collagenase I (SCR103), 250 U/mL collagenase XI (C7657), 120 U/mL hyaluronidase (H3506) (Sigma-Aldrich), and 120 U/mL DNAse I (10104159001, Roche) for 45 minutes, followed by quenching with RPMI 1640 plus 10% fetal bovine serum, after which they were passed through a 70 μm cell strainer. Pellets were washed again then resuspended with LIVE/DEAD Aqua Stain (L34957; Thermo Fisher Scientific), followed by addition of FCγR block (101320; BioLegend), flow cytometry buffer (FACS buffer), 1× PBS (Ca^2+^ and Mg^2+^ free) containing 5% FBS, and 5 mM EDTA for 15 minutes, followed by addition of antibody cocktail on ice for 30 minutes.

Cells were fixed and permeabilized (554714, BD Bioscience) to stain intracellular antigens according to the manufacturer’s instructions. Cells were washed with FACS buffer 2 times and then resuspended in 200 μl FACS buffer. Flow cytometry was performed on a Bio-Rad Ze5 equipped with 405 nm, 488 nm, 561 nm, and 640 nm lasers using Everest software, version 2.

Data and compensation were analyzed with FlowJo software (FlowJo 10.8.1, BD). Antibodies used in flow cytometry were as follows: FITC anti-CD45 (103108, 10 μg/mL), PE-Cy7 anti-CD11b (101216, 5 μg/mL), BV421 anti–Ly-6C (128031, 4 μg/mL), BV605 Ly-6G (127639, 6 μg/mL), APC-Fire750 anti-CCR2 (150630, 8 μg/mL), BV785 anti-F4/80 (123141, 5 μg/mL), AF700 anti-MHCII (107622, 4μg/mL) (all from BioLegend), and PE anti-uPAR (FAB531P, Bio-Techne, 1 μg/mL).

#### Monocyte migration assay.

The spleens of C57BL/6J WT mice and *suPAR^Tg^* mice were mechanically disrupted through a 70 μm cell strainer, and splenic monocytes were isolated using The Mouse Monocyte Negative Selection Kit (19861; STEMCELL). The chemotaxis ability of isolated monocytes was assessed by cell migration assay (CBA-105; Cell BioLabs) according to the product manual. Briefly, the monocyte suspension was added to the upper membrane chamber. The bottom tray contained chemoattractant solution and RPMI media with or without 1000 ng/ml CCL2 (PHC1011; Life Technologies Corp.). After 4 and 8 hours, both the cells adherent to the membrane and cells in the bottom tray were collected and stained by CyQUANT dye (C7026, Thermo Fisher Scientific). Fluorescence measurement was performed with a 485/538 nm filter set and a 530 nm cutoff.

#### Statistics.

All results are presented as mean ± SEM. Comparisons between multiple groups were performed with Student’s *t* test, 1-way ANOVA, and 2-way ANOVA with Tukey’s multiple comparison test, where appropriate. A 2-tailed *P* value of less than 0.05 was considered significant. GraphPad Prism was used to perform statistical analysis and to generate figures.

#### Study approval.

All participants gave written informed consent for their respective studies, and the study protocols were approved by the Institutional Review Board at each participating Clinical Coordinating Center. Animal experiments were carried out with approval of the University of Michigan Institutional Animal Care and Use Committee.

## Author contributions

SSH, KCD, and DRG designed the research study. GH, DJT, AV, CW, FP, HW, ABO, SG, GHH, JD, ACF, KMA, GKE, AT, AI, NS, JSP, LWT, CE, OBP, KB, SB, HU, JEO, SRO, MEH, JBN, LAL, GE, OM, MOM, VLM, DJP, CJW, JR, DRG, KCD, SSH, LZ, and SRH contributed to the collection, analysis, and interpretation of data. DJT, CW, FP, HW, PB, KMA, GKE, AT, AI, CL, and GH conducted laboratory experiments. GH, DJT, AV, and SSH wrote the first draft. All authors reviewed the initial draft and provided critical revisions. All authors reviewed, edited, and approved the final version of the manuscript. Order of co–first authors was determined based on alphabetical order of surnames.

## Supplementary Material

Supplemental data

## Figures and Tables

**Table 1:**
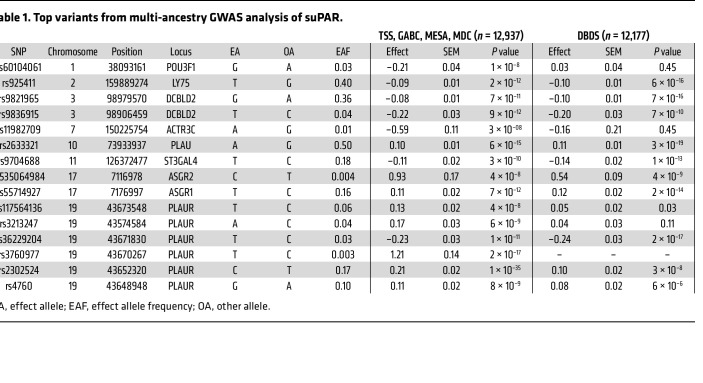
Top variants from multi-ancestry GWAS analysis of suPAR.

## Version 1 10/04/2022

In-Press Preview

## Version 2 12/15/2022

Electronic publication
