# Supplementary material for: Increased soluble urokinase plasminogen activator levels modulate monocyte function to promote atherosclerosis
Source: J Clin Invest. 2022 Dec 15;132(24):e158788. doi: 10.1172/JCI158788 (PMC9754000; doi:10.1172/JCI158788)
Supplement: Supplemental data [file jci-132-158788-s008.pdf]

Supplementary Appendix

I. **Page 2 –** Members of the DBDS Genomic Consortium

II. **Page 3 –** Regeneron Genetics Center Banner Author List and Contribution Statements

III. **Page 5** – Figure S1: Adjusted mean CAC score at baseline and follow-up by suPAR categories

IV. **Page 6** – Figure S2: Adjusted Kaplan-Meier cumulative incidence of any cardiovascular disease event by suPAR categories

V. **Page 7** – Figure S3: Association between baseline suPAR and incidence of any cardiovascular disease event

VI. **Page 8** – Figure S4: Distribution of suPAR levels by cohort

VII. **Page 9** – Figure S5: Manhattan plot for genome-wide associations with suPAR in multi-ancestry and European ancestry

VIII. **Page 10** – Figure S6: UPAR protein sequence and missense variant annotation

IX. **Page 11-14** – Figure S7: Regional plots for loci associated with suPAR from European-ancestry analysis

X. **Page 15-16** – Figure S8: Regional plots in the PLAUR locus after sequential conditional analysis on top variants

XI. **Page 17 –** Figure S9: PLAUR gene expression and uPAR cellular distribution in HEK cells

XII. **Page 18 –** Figure S10: Immunostaining of murine liver tissue showing successful production of suPAR

XIII. **Page 19 –** Figure S11: Mendelian randomization of genetically predicted suPAR by rs4760 with CVD in CARDIoGRAM and MVP

XIV. **Page 20 –** Figure S12: Mendelian randomization phenome-wide association of genetically predicted suPAR by rs2302524 with CVD and rare damaging missense variants' impact on the odds of ischemic heart disease

XV. **Page 21 –** Figure S13: Association of suPAR with creatinine-derived eGFR and CKD as predicted by rs4760 PLAUR missense variant in CKDGen and UKB

XVI. **Page 22** – Figure S14: Cholesterol and suPAR levels in wild-type and transgenic mice prior to and 10 weeks after PSCK9-AAV transfection

XVII. **Page 23** – Figure S15: Cholesterol, atherosclerotic lesion and necrotic sinus area in wild-type and transgenic mice over-expressing suPAR stratified by sex (male n=11, female n=7)
XVIII. **Page 24 –** Figure S16: SuPAR is detectable in both wild-type and suPAR-Tg atherosclerotic plaque
XIX. **Page 25 –** Figure S17: Bone marrow-derived macrophages exhibit a pro-atherogenic phenotype compared to WT bone marrow-derived macrophages
XX. **Page 26** – Figure S18: QQ plots for genome-wide associations with suPAR
XXI. **Page 27 –** Figure S19: Chromatogram by Sanger sequencing of PLAUR reference allele and variants
XXII. **Page 28** – Table S1: Baseline characteristics of MESA cohort by suPAR categories
XXIII. **Page 29** – Table S2: Association between baseline CAC score and suPAR levels
XXIV. **Page 30** – Table S3: Longitudinal association between baseline suPAR levels and CAC score during follow-up
XXV. **Page 31 –** Table S4: Survival analysis for baseline suPAR levels and CVD events
XXVI. **Page 32 –** Table S5: Survival analysis for baseline suPAR levels and CVD events in MESA with time-varying eGFR
XXVII. **Page 33 –** Table S6: Multivariable survival analysis for baseline suPAR levels and CVD events in MESA with high-sensitivity troponin T and NT-proBNP
XXVIII. **Page 34** – Table S7: Top variants from genome-wide association analysis of suPAR in participants with European ancestry
XXIX. **Page 35** – Table S8: Finemapped signals of the PLAUR locus using SuSie.
XXX. **Page 36-37** – Table S9: UK Biobank Disease Phenotype Definitions
XXXI. **Page 38** – References

Members of the DBDS Genomic Consortium

Steffen Andersen[1]
Karina Banasik[2]
Søren Brunak[2]
Kristoffer Burgdorf[3]
Christian Erikstrup[4]
Thomas Folkmann Hansen[5]
Henrik Hjalgrim[6]
Gregor Jemec[7]
Poul Jennum[8]
Pär Ingemar Johansson[3]
Kasper Rene Nielsen[9]
Mette Nyegaard[10]
Mie Topholm Brun[11]
Ole Birger Pedersen[12]
Susan Mikkelsen[13]
Khoa Manh Dinh[13]
Erik Sørensen[3]
Henrik Ullum[3]
Sisse Rye Ostrowski[3]
Thomas Werge[14]
Daniel Gudbjartsson[15]
Kari Stefansson[15]
Hreinn Stefánsson[15]
Unnur Þorsteinsdóttir[15]
Margit Anita Hørup Larsen[3]
Maria Didriksen[3]
Susanne Sækmose[16]

[1] Department of Finance Copenhagen Business School Copenhagen Denmark
[2] Novo Nordisk Foundation Center for Protein Research Faculty of Health and Medical Sciences University of Copenhagen Copenhagen Denmark
[3] Department of Clinical Immunology Copenhagen University Hospital Copenhagen Denmark
[4] Department of Clinical Immunology Aarhus University Hospital Aarhus Denmark
[5] Danish Headache Center Department of Neurology Rigshospitalet Glostrup Denmark
[6] Department of Epidemiology Research Statens Serum Institut Copenhagen Denmark
[7] Department of Clinical Medicine Zealand University Hospital Roskilde Denmark
[8] Department of Clinical Neurophysiology at University of Copenhagen Copenhagen Denmark
[9] Department of Clinical Immunology Aalborg University Hospital Aalborg Denmark
[10] Department of Biomedicine Aarhus University Denmark
[11] Department of Clinical Immunology Odense University Hospital Odense Denmark
[12] Department of Clinical Immunology Zealand University Hospital, Køge Denmark
[13] Department of Clinical Immunology Aarhus University Hospital Aarhus Denmark
[14] Institute of Biological Psychiatry Mental Health Centre Sct. Hans Copenhagen University Hospital Roskilde Denmark
[15] deCODE genetics Reykjavik Iceland
[16] Department of Clinical Immunology, Zealand University Hospital Køge Denmark

Regeneron Genetics Center Banner Author List and Contribution Statements

RGC Management and Leadership Team
Goncalo Abecasis, D.Phil. , Aris Baras, M.D. , Michael Cantor, M.D. , Giovanni Coppola, M.D. , Andrew Deubler , Aris Economides, Ph.D. , Katia Karalis, Ph.D. , Luca A. Lotta, M.D., Ph.D. , John D. Overton, Ph.D. , Jeffrey G. Reid, Ph.D. , Katherine Siminovitch, M.D. , Alan Shuldiner, M.D.

Sequencing and Lab Operations
Christina Beechert , Caitlin Forsythe, M.S. , Erin D. Fuller , Zhenhua Gu, M.S. , Michael Lattari , Alexander Lopez, M.S., John D. Overton, Ph.D. , Maria Sotiropoulos Padilla, M.S. , Manasi Pradhan, M.S. , Kia Manoochehri, B.S. , Thomas D. Schleicher, M.S. , Louis Widom , Sarah E. Wolf, M.S. , Ricardo H. Ulloa, B.S.

Clinical Informatics
Amelia Averitt, Ph.D. , Nilanjana Banerjee, Ph.D. , Michael Cantor, M.D. , Dadong Li, Ph.D. , Sameer Malhotra, M.D. , Deepika Sharma, MHI , Jeffrey Staples , Ph.D.

Genome Informatics
Xiaodong Bai, Ph.D. , Suganthi Balasubramanian, Ph.D. , Suying Bao, Ph.D. , Boris Boutkov, Ph.D. , Siying Chen, Ph.D. , Gisu Eom, B.S. , Lukas Habegger, Ph.D. , Alicia Hawes, B.S. , Shareef Khalid , Olga Krasheninina, M.S. , Rouel Lanche, B.S. , Adam J. Mansfield, B.A. , Evan K. Maxwell, Ph.D. , George Mitra, B.A. , Mona Nafde, M.S. , Sean O'Keeffe, Ph.D. , Max Orelus, B.B.A. , Razvan Panea, Ph.D. , Tommy Polanco, B.A. , Ayesha Rasool, M.S. , Jeffrey G. Reid, Ph.D. , William Salerno, Ph.D. , Jeffrey C. Staples, Ph.D. , Kathie Sun, Ph.D. , Jiwen Xin, Ph.D.

Analytical Genomics and Data Science
Goncalo Abecasis, D.Phil. , Joshua Backman, Ph.D. , Amy Damask, Ph.D. , Lee Dobbyn, Ph.D. , Manuel Allen Revez Ferreira, Ph.D. , Arkopravo Ghosh, M.S. , Christopher Gillies, Ph.D. , Lauren Gurski, B.S. , Eric Jorgenson, Ph.D. , Hyun Min Kang, Ph.D. , Michael Kessler, Ph.D. , Jack Kosmicki, Ph.D. , Alexander Li , Ph.D. , Nan Lin, Ph.D. , Daren Liu, M.S. , Adam Locke, Ph.D. , Jonathan Marchini, Ph.D. , Anthony Marcketta, M.S. , Joelle Mbatchou, Ph.D. , Arden Moscati, Ph.D. , Charles Paulding, Ph.D. , Carlo Sidore, Ph.D. , Eli Stahl, Ph.D. , Kyoko Watanabe, Ph.D. , Bin Ye, Ph.D. , Blair Zhang, Ph.D. , Andrey Ziyatdinov, Ph.D.

Therapeutic Area Genetics
Ariane Ayer, B.S. , Aysegul Guvenek, Ph.D. , George Hindy, Ph.D. , Giovanni Coppola, M.D. , Jan Freudenberg, M.D. , Jonas Bovijn M.D. , Julie Horowitz, Ph.D. , Katherine Siminovitch, M.D. , Jonas B. Nielsen, MD, PhD, Kavita Praveen, Ph.D. , Luca A. Lotta, M.D. , Manav Kapoor, Ph.D. , Mary Haas, Ph.D. , Moeen Riaz , Ph.D. , Niek Verweij, Ph.D. , Olukayode Sosina, Ph.D. , Parsa Akbari, Ph.D. , Priyanka Nakka, Ph.D. , Sahar Gelfman, Ph.D. , Sujit Gokhale, B.E. , Tanima De, Ph.D. , Veera Rajagopal, Ph.D. , Alan Shuldiner, M.D. , Bin Ye, Ph.D. , Gannie Tzoneva, Ph.D. , Juan Rodriguez-Flores, Ph.D.

RGC Biology
Shek Man Chim, Ph.D. , Valerio Donato, Ph.D. , Aris Economides, Ph.D. , Daniel Fernandez, M.S. , Giusy Della Gatta, Ph.D. , Alessandro Di Gioia, Ph.D. , Kristen Howell, M.S. , Katia Karalis, Ph.D. , Lori Khrimian, Ph.D. , Minhee Kim, Ph.D. , Hector Martinez , Lawrence Miloscio, B.S. , Sheilyn Nunez, B.S. , Elias Pavlopoulos, Ph.D. , Trikaldarshi Persaud, B.S.

Research Program Management & Strategic Initiatives
Esteban Chen, M.S. , Marcus B. Jones, Ph.D. , Michelle G. LeBlanc, Ph.D. , Jason Mighty, Ph.D. , Lyndon J. Mitnaul, Ph.D. , Nirupama Nishtala, Ph.D. , Nadia Rana, Ph.D.

Figure S1. Adjusted mean change in CAC score between baseline and follow-up by suPAR categories

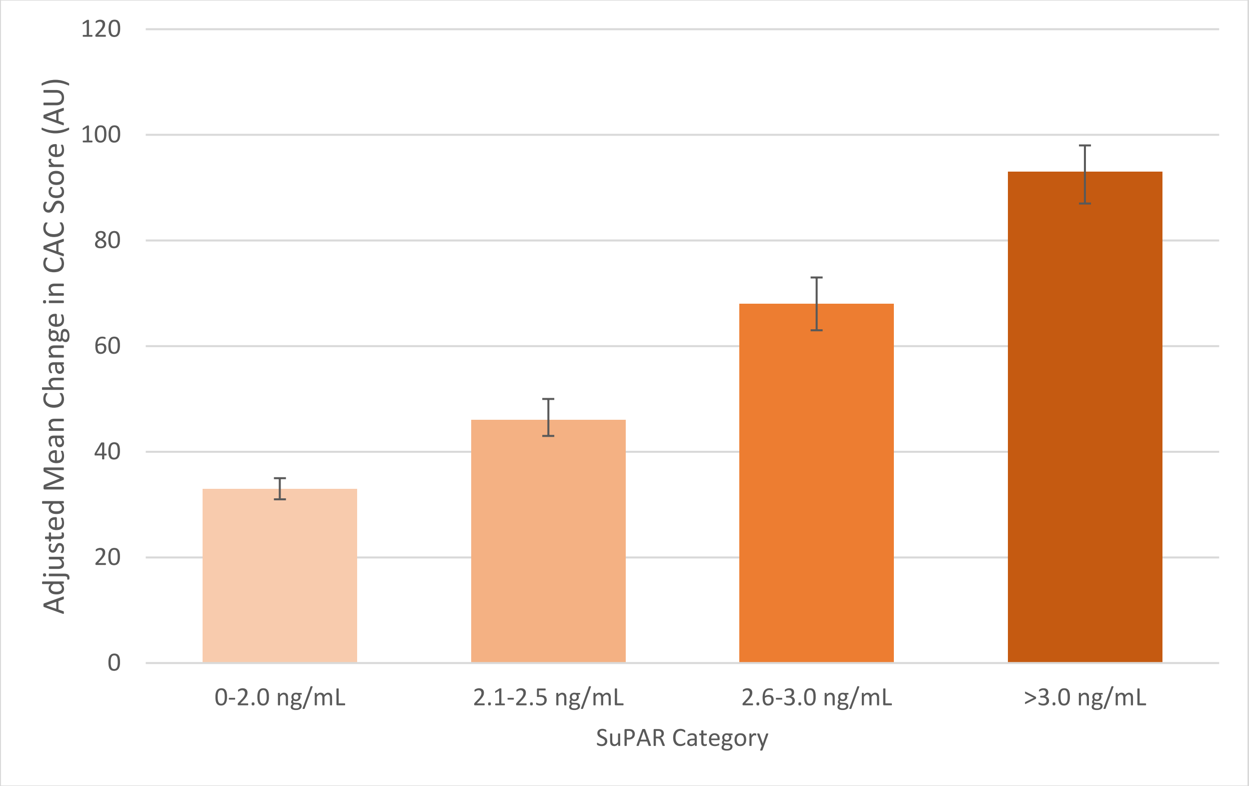

Adjusted mean change in CAC score (Agaston units [AU]) based on Agaston scoring method between baseline and initial follow-up visit (follow-up minus baseline) stratified by suPAR categories: 0-2.0 ng/mL, 2.0-2.5 ng/mL, 2.5-3.0 ng/mL, and >3.0 ng/mL. Mean CAC score for each category based on the predicted CAC score values accounting for age, sex, race, body-mass index, history of smoking, estimated glomerular filtration rate, low density lipoprotein levels, high density lipoprotein levels, C-reactive protein, and diabetes mellitus. Error bars represent 95% confidence intervals. Abbreviations: CAC, coronary artery calcium; MESA, Multi-Ethnic Study of Atherosclerosis; suPAR, soluble urokinase plasminogen activator receptor

Figure S2. Adjusted Kaplan-Meier cumulative incidence of any cardiovascular disease event by suPAR categories

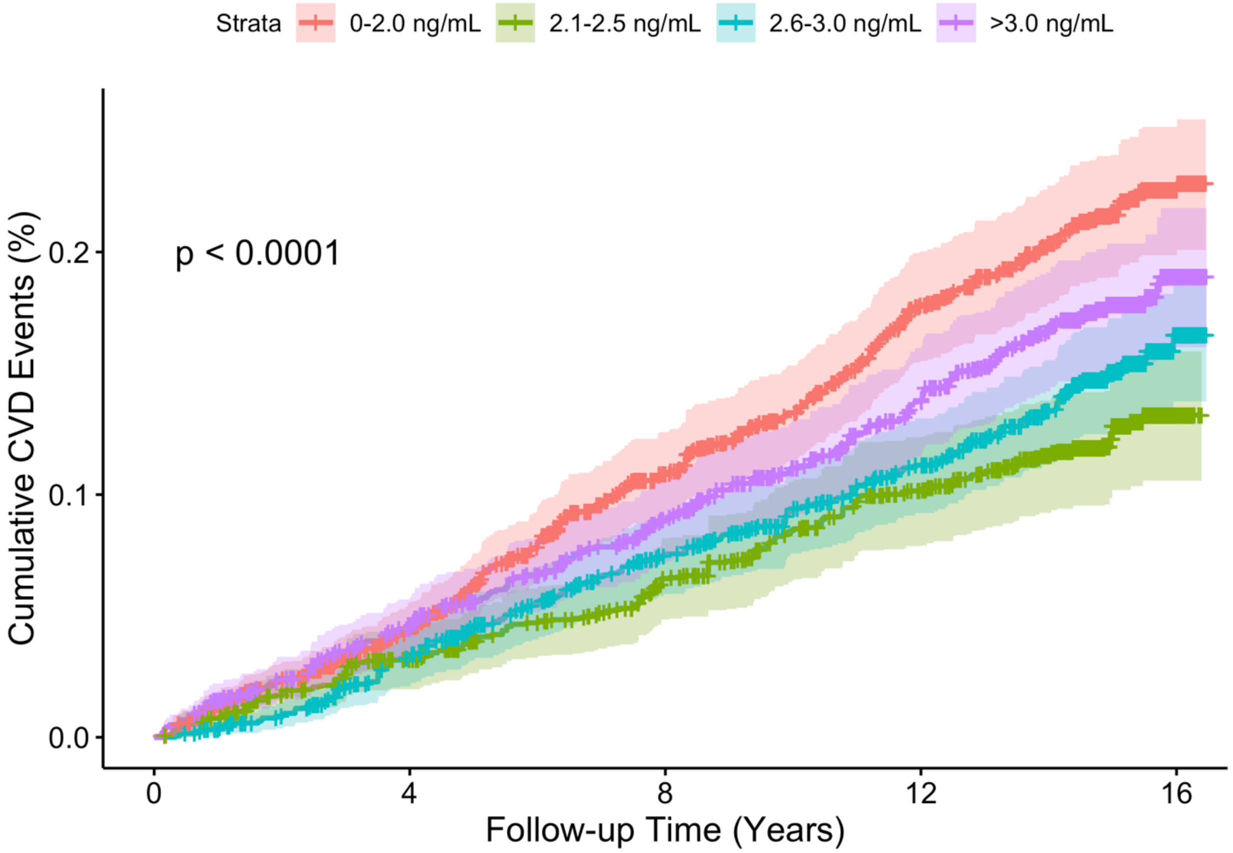

Inverse-probability weighted Kaplan-Meier curves for the cumulative incidence of cardiovascular disease (CVD) events stratified by suPAR categories: 0-2.0 ng/mL (red), 2.0-2.5 ng/mL (green), 2.5-3.0 ng/mL (blue), >3 ng/mL (purple). Propensity scores were estimated for suPAR categories using generalized boost models adjusted for age, sex, race, body-mass index, history of smoking, estimated glomerular filtration rate, low density lipoprotein levels, high density lipoprotein levels, C-reactive protein, and diabetes mellitus. The difference in cumulative incidence curves between suPAR categories was tested using the log-rank test. A CVD event was defined as the composite of myocardial infarction, resuscitated cardiac arrest, angina, revascularization, stroke (excluding transient ischemic attack), or death due to CVD.

Figure S3. Association between baseline suPAR (per 100% increase) and incidence of any cardiovascular disease event

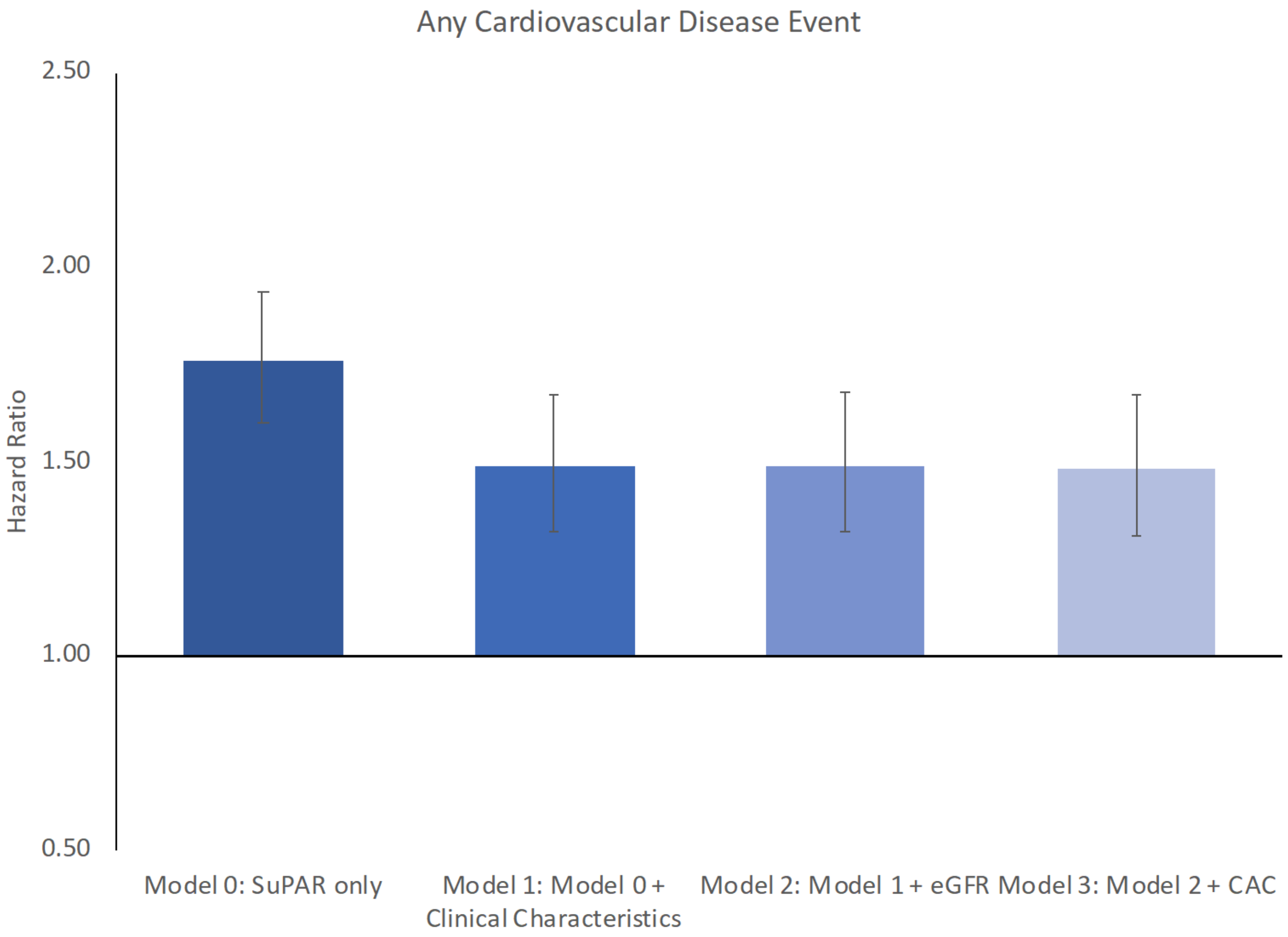

Bar graphs showing the hazard ratio (represented by height of the bar) and 95% confidence intervals (represented by error bars) for the association of suPAR (per 100%) and incidence of cardiovascular disease. Model 0: suPAR only; Model 1: suPAR and age, sex, race, body-mass index, history of smoking, low-density lipoprotein, high-density lipoprotein, C-reactive protein, hypertension, and diabetes mellitus; Model 2: Model 1 + baseline estimated glomerular filtration rate; Model 3: Model 2 + baseline coronary artery calcification (CAC) score. A CVD event was defined as the composite of myocardial infarction, resuscitated cardiac arrest, angina, revascularization, stroke (excluding transient ischemic attack), or death due to CVD.

Figure S4. Distribution of suPAR levels by cohort

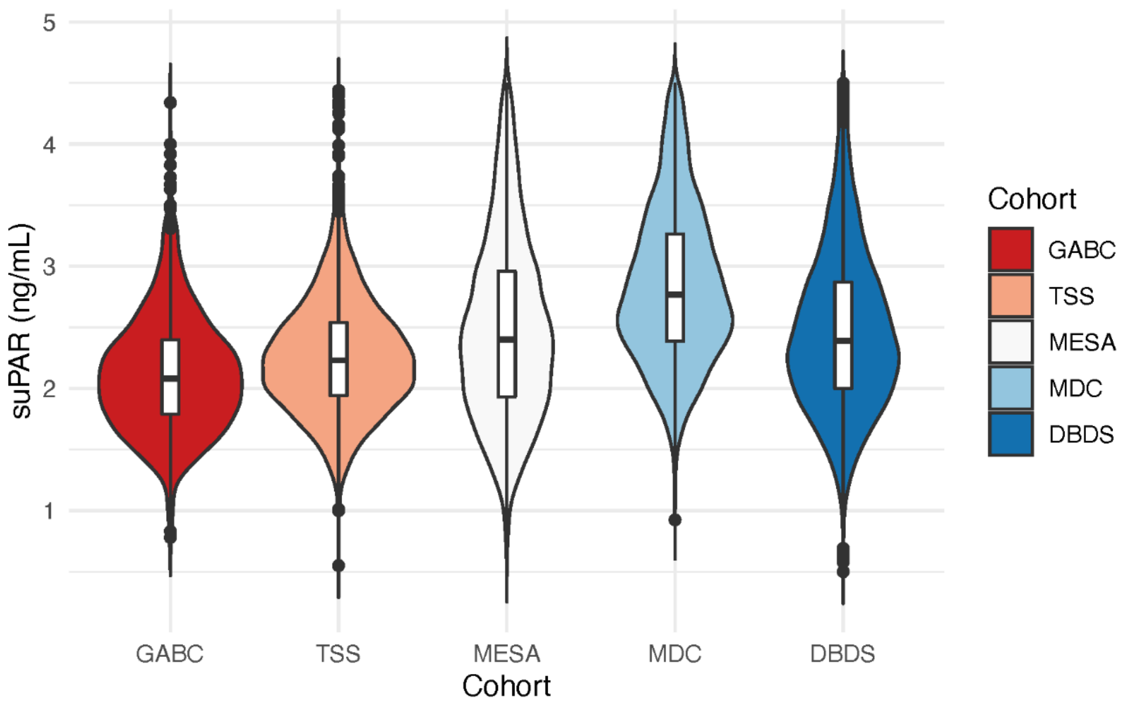

Violin and box plot for suPAR levels in each of the four main cohorts that are included in the overall meta-analysis and in the replication cohort. suPAR levels below 0.5 ng/mL and above 4.5 ng/mL were considered as outliers and excluded for the purposes of visualization. All the data including outliers were included in the analyses.

Abbreviations: GABC, Genes and Blood-Clotting study; TSS, The Trinity Student Study; MESA, Multi-Ethnic Study of Atherosclerosis; MDC, The Malmö Diet and Cancer study.

Figure S5. Manhattan plot for genome-wide associations with suPAR in multi-ancestry and European ancestry analyses

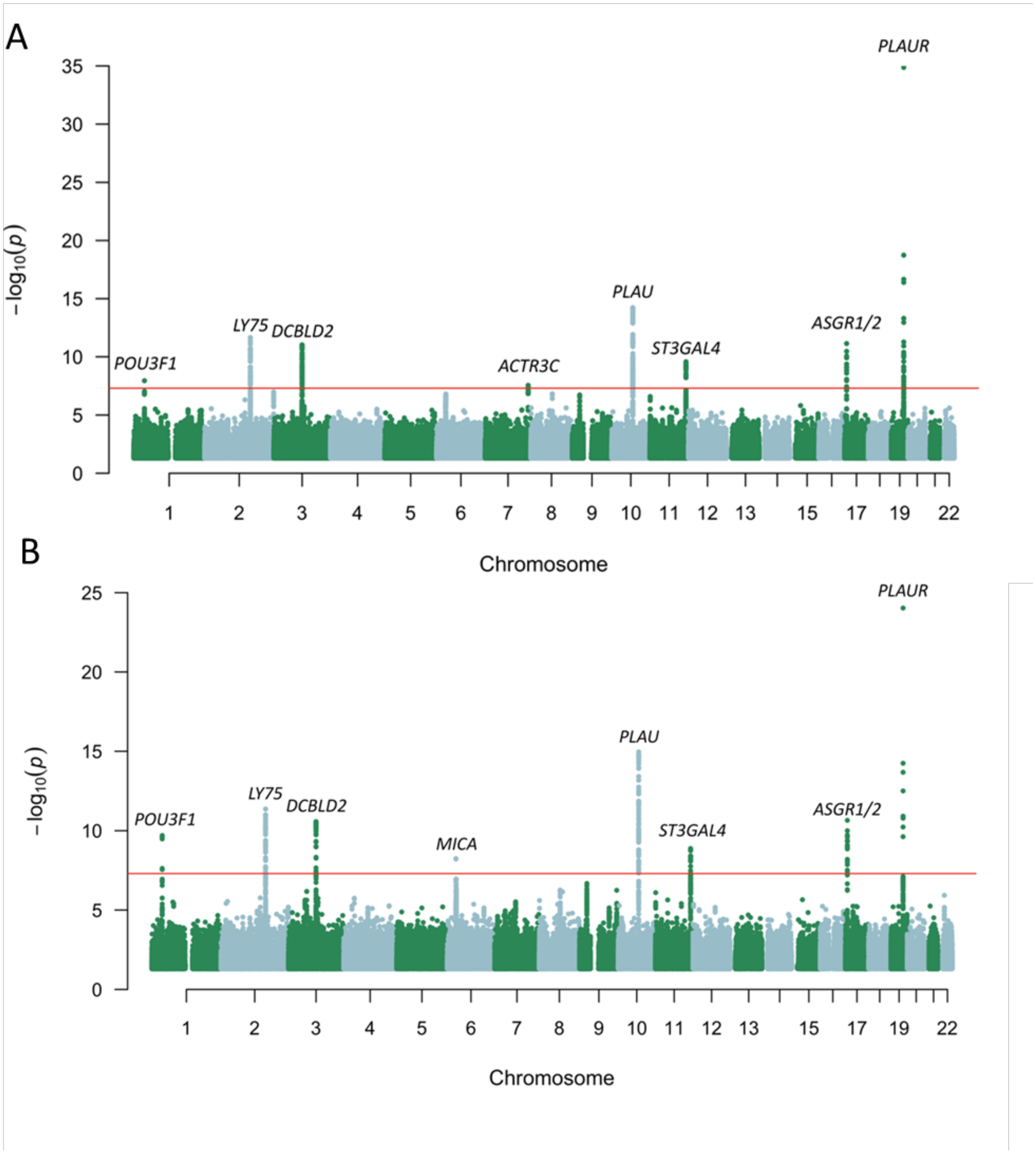

Manhattan plots for genome-wide associations with suPAR. Plot of the minus log10 of the P-values observed in the genome-wide meta-analysis of suPAR levels in multi-ancestry (A) and European ancestry (B) samples. Significant ($P<5\times10^{-8}$) signals were observed with variants in 8 genomic locations for both meta-analyses. The signal in the *ACTR3C* locus was specific to multi-ancestry analysis while the signal in *MICA* was specific to European ancestry analysis.

Figure S6. UPAR protein sequence and missense variant annotation

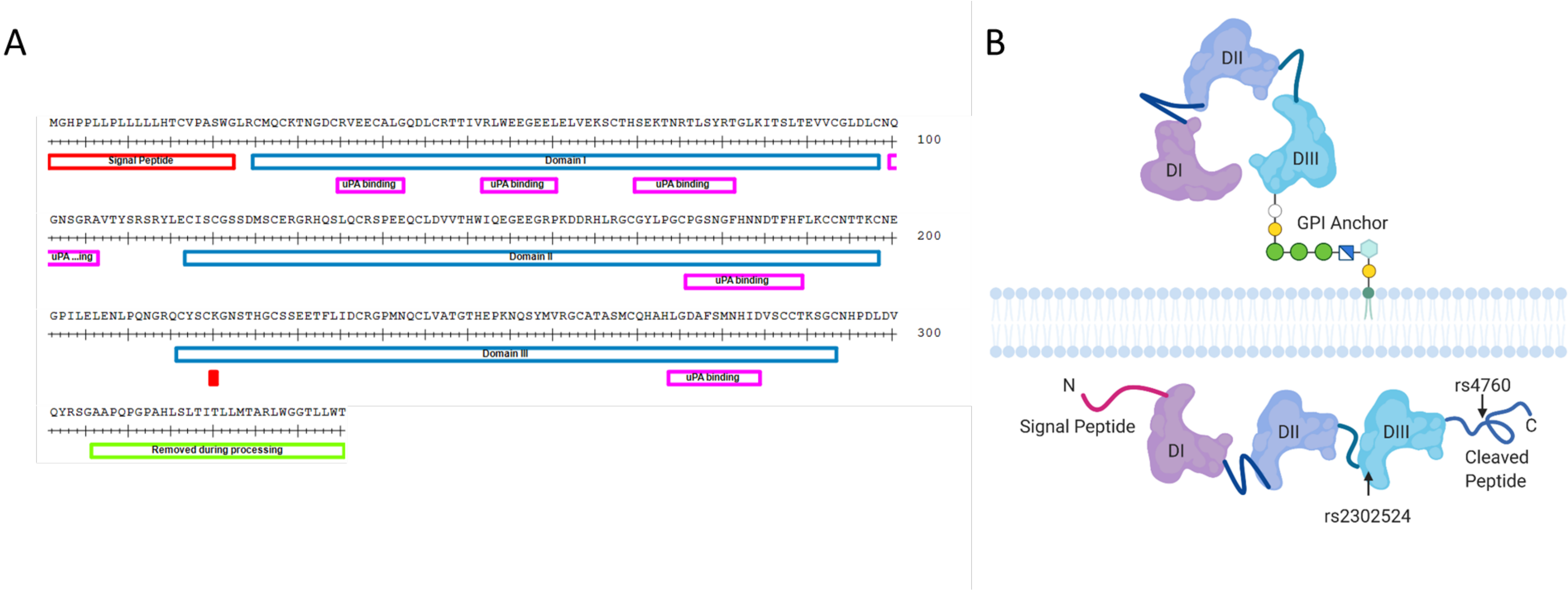

Panel (A) The linear amino acid sequence of the full-length transcript was obtained from Ensembl. Domain annotations and uPA binding domains were annotated using previously published data.[1] (B) Representative protein diagram where approximate location of missense mutations on preprotein is indicated with rs number and arrow. Images were generated using BioRender, www.biorender.com.

Figure S7. Regional plots for loci associated with suPAR from European-ancestry analysis

A. *PLAUR*

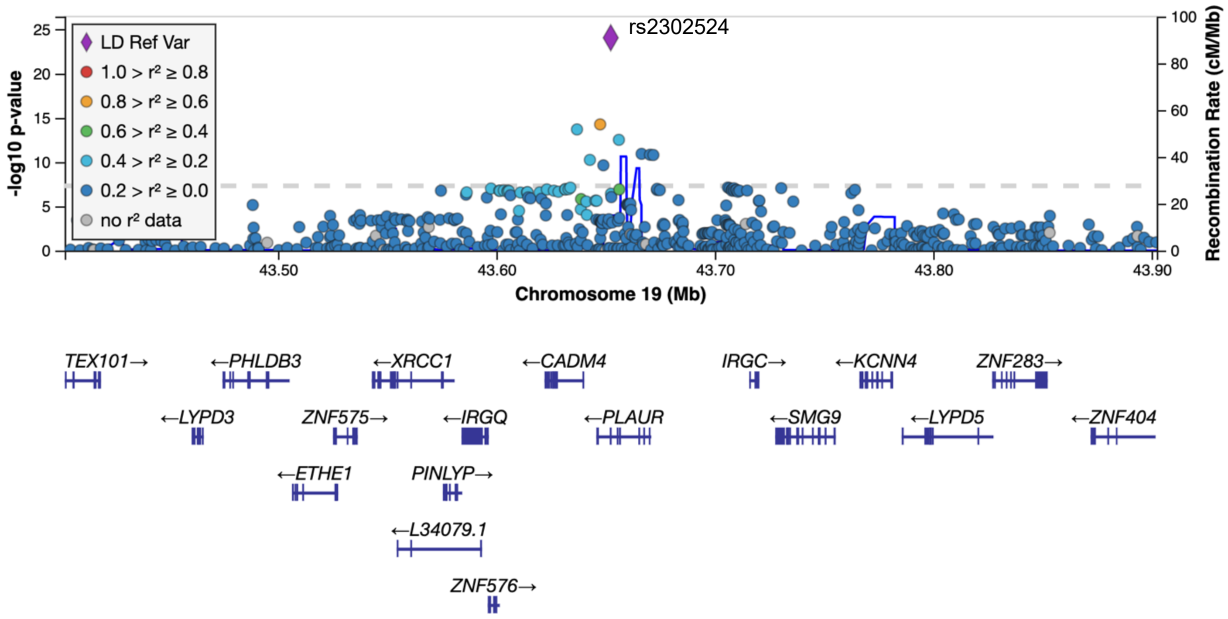

B. *PLAU*

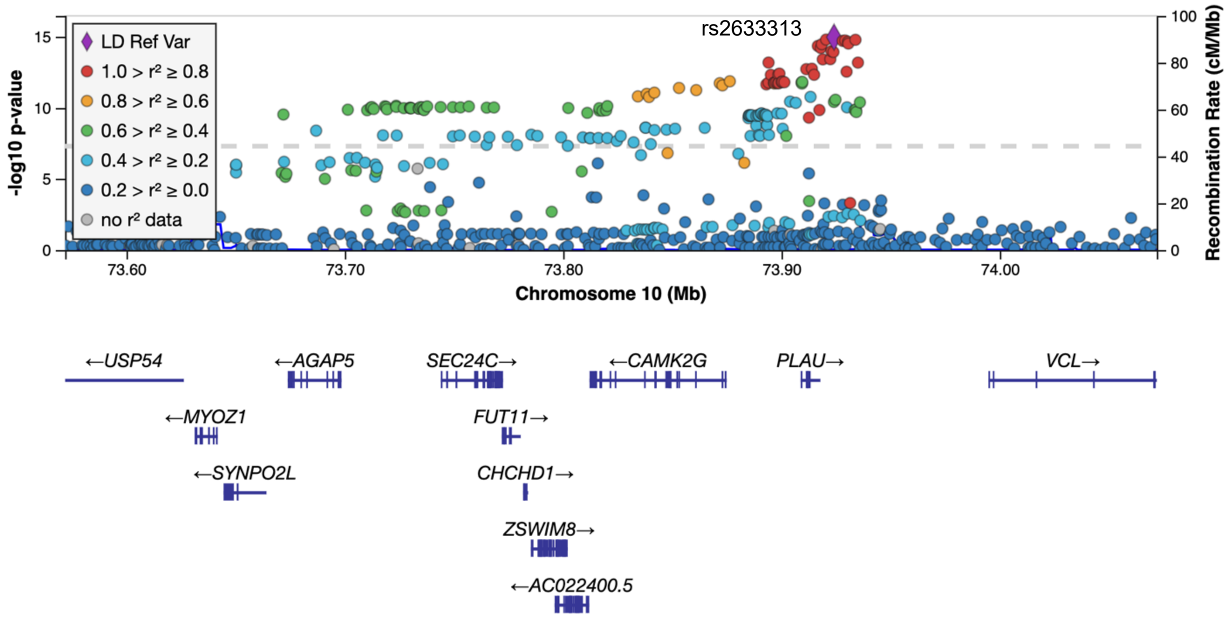

C. *LY75*

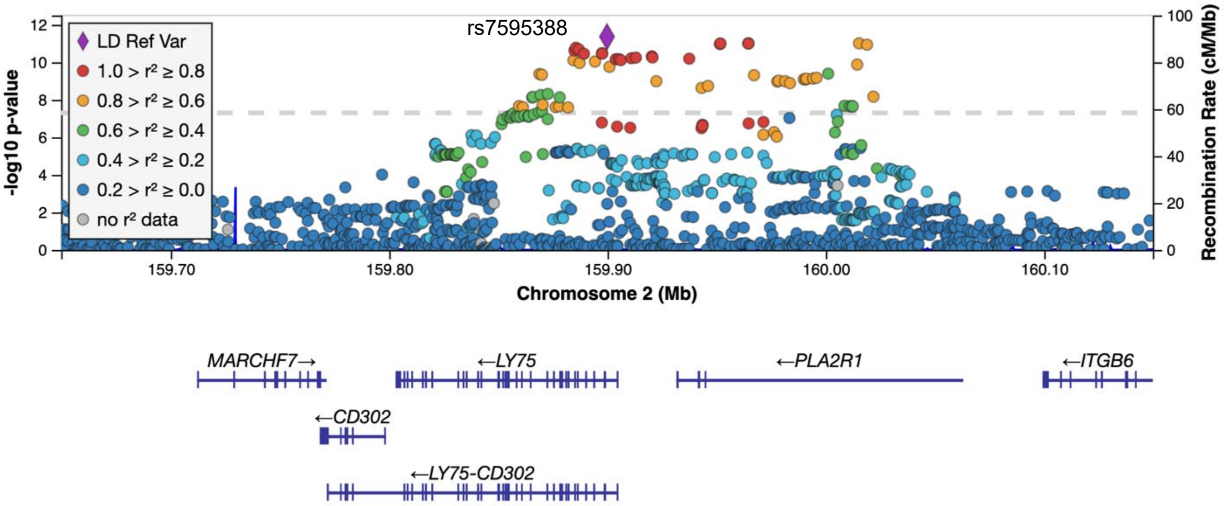

D. *ASGR1; ASGR2*

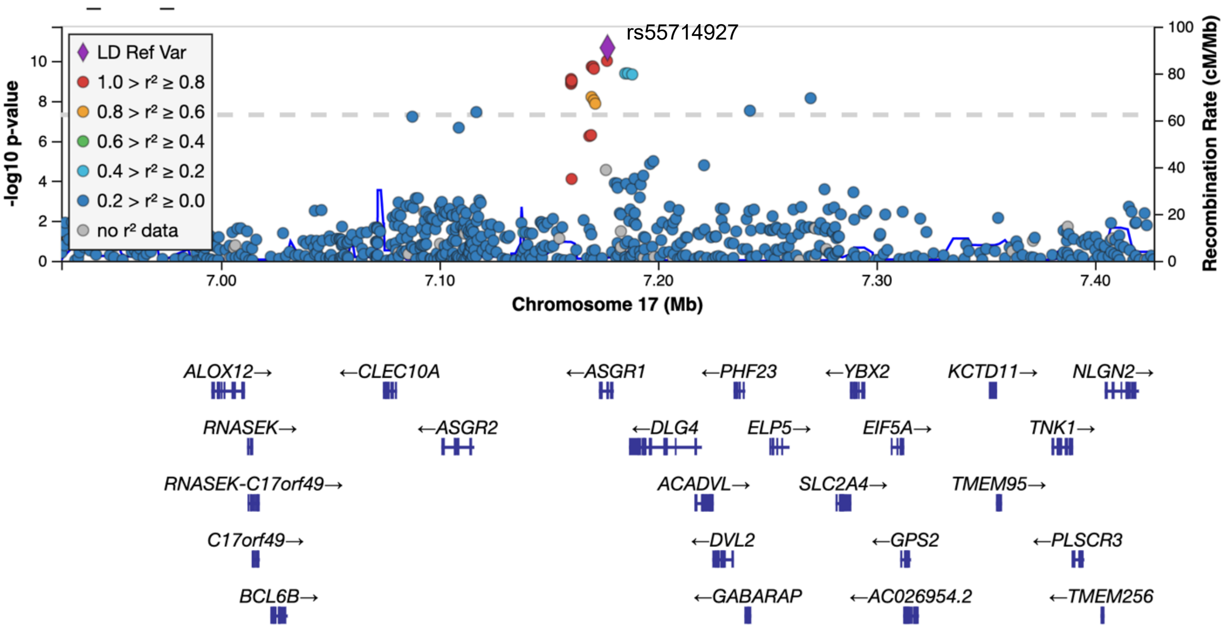

E. ***DCBLD2/ST3GAL6***

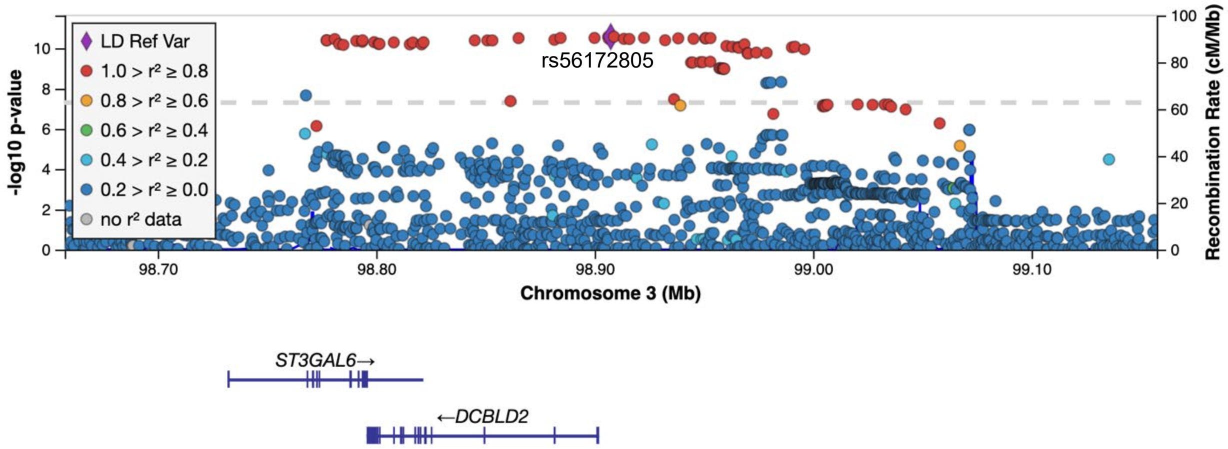

F. ***POU3F1***

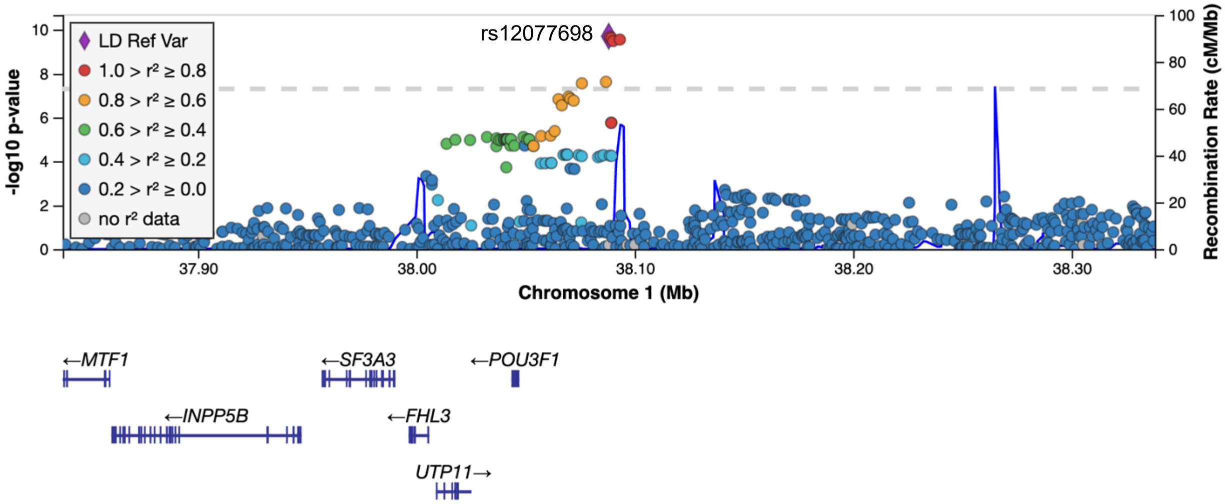

G. ***ST3GAL4***

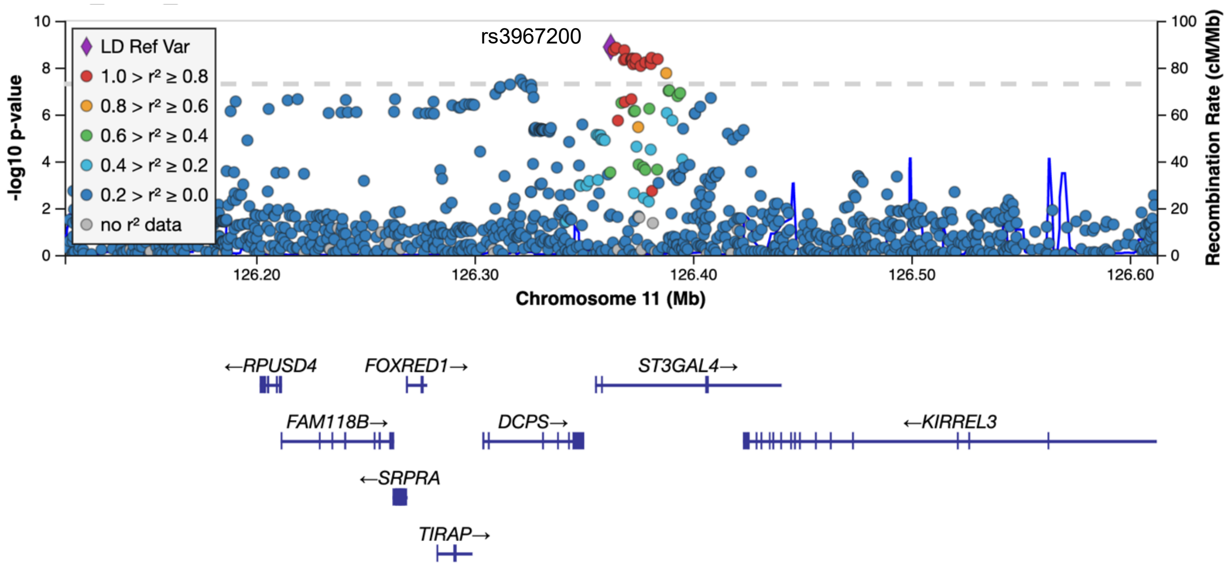

H. ***MICA***

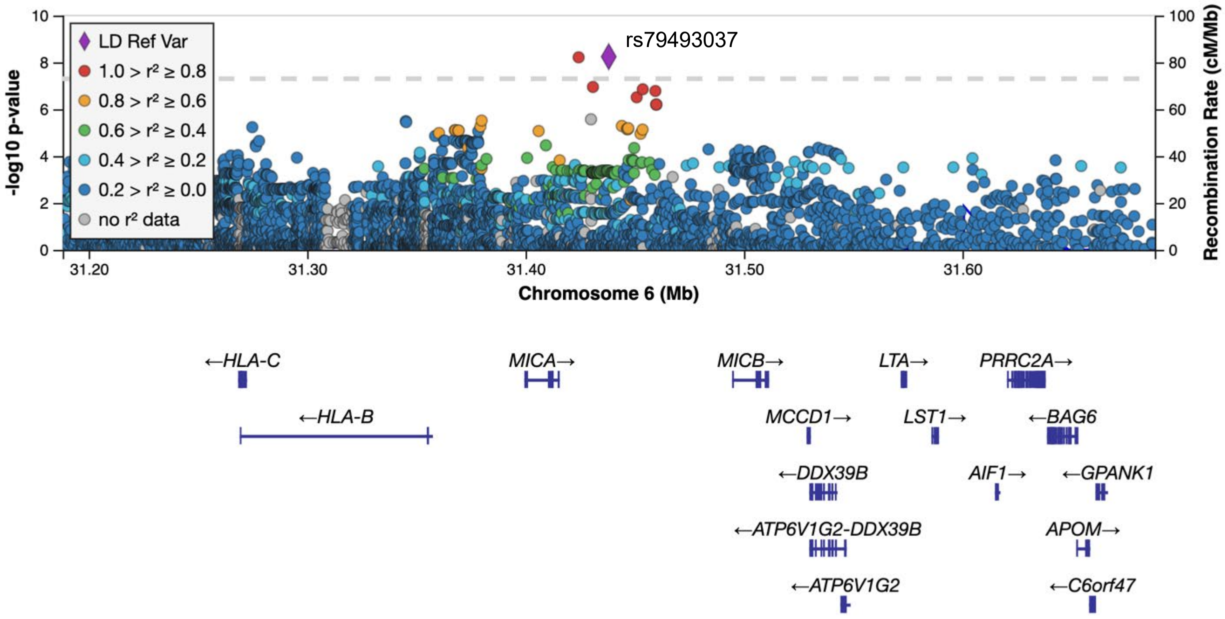

Plots of the minus log10 of the P-values observed in the genome-wide meta-analysis of suPAR levels in European ancestry of variants in each of the genome-wide significant loci. The horizontal dashed line was drawn at P = 5 × 10-8. The different colors denote the correlation with the variant with the lowest P value in each locus. The regional plots were drawn using LocusZoom.

Figure S8. Regional plots in the *PLAUR* locus after sequential conditional analysis on top variants

A. Conditional on rs2302524

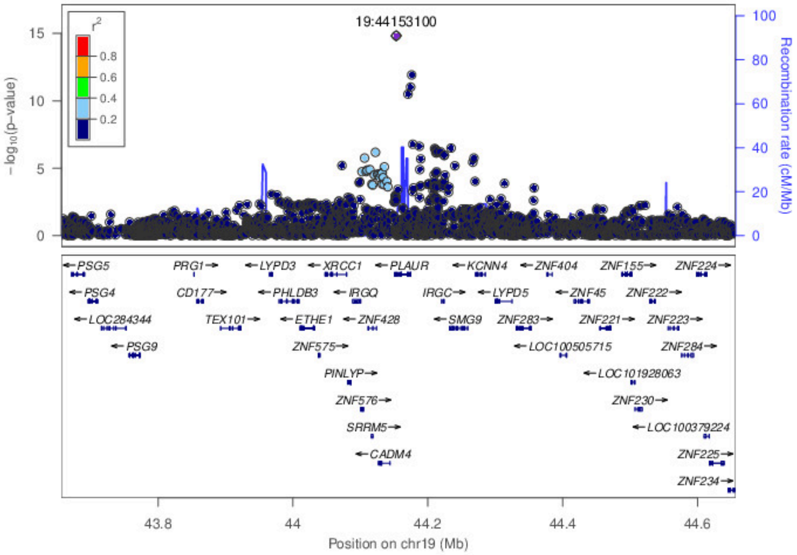

B. Conditional on rs2302524 and rs4760

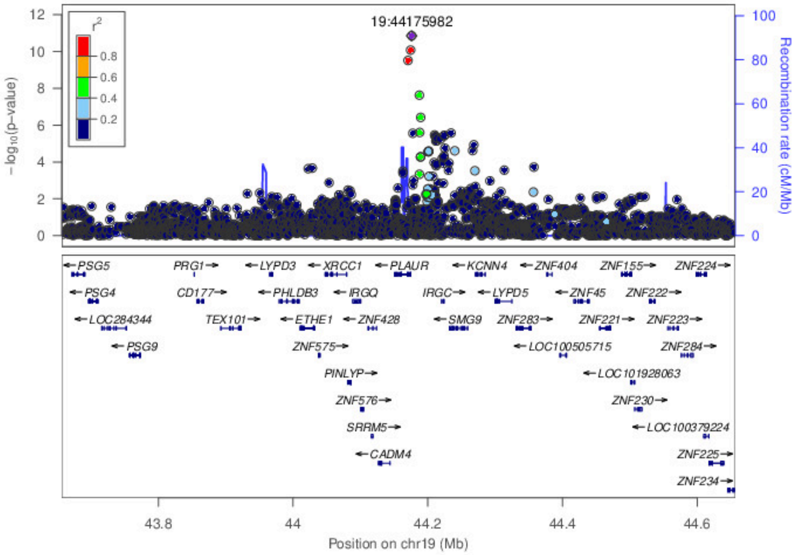

C. Conditional on rs2302524, rs4760, and rs36229204

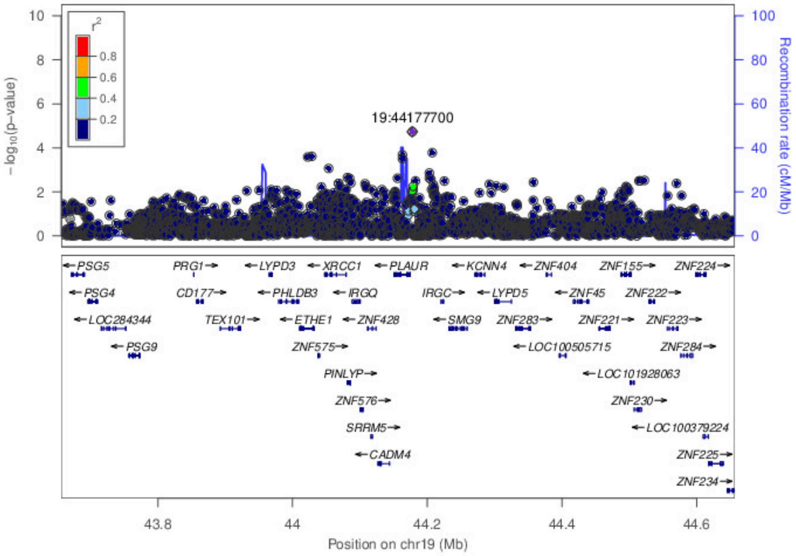

Figure S9: PLAUR gene expression and uPAR cellular distribution in HEK cells

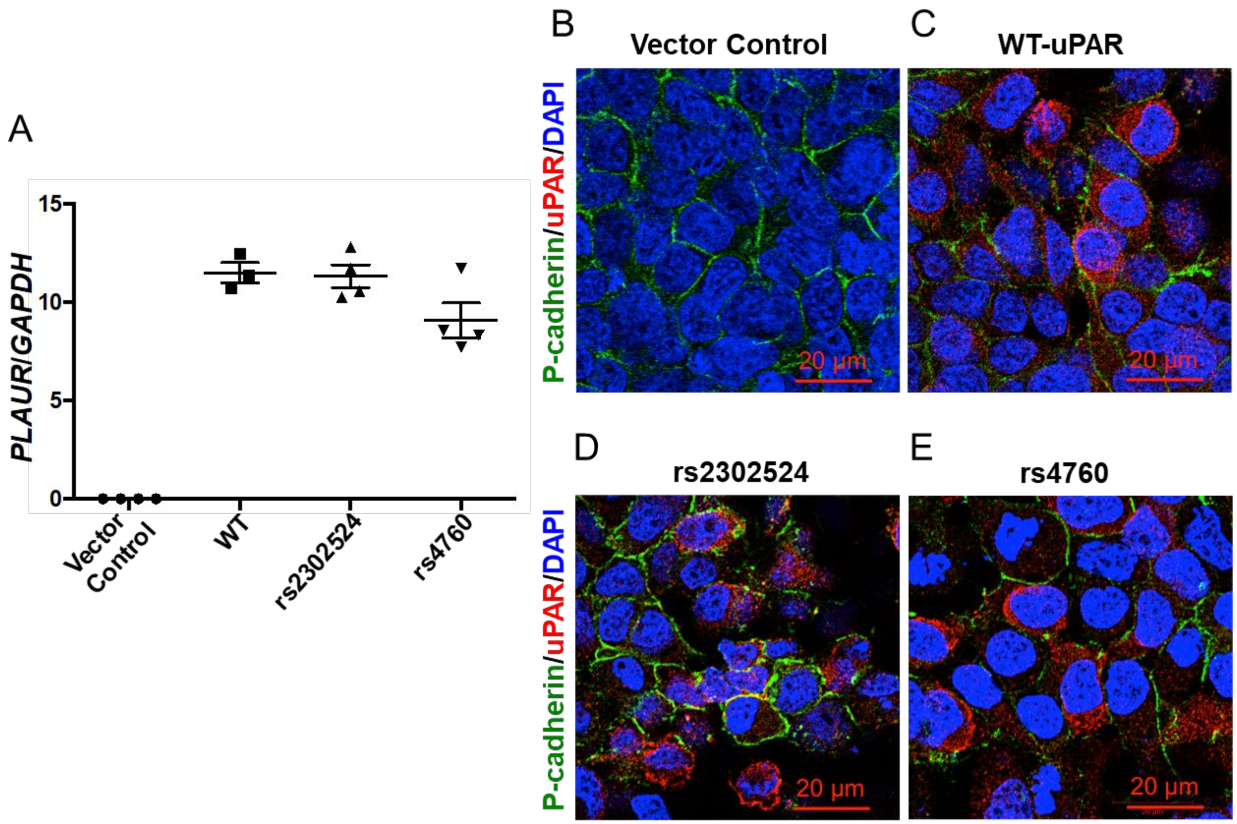

Panel A shows gene expression levels using real-time quantitative polymerase chain reaction analysis of uPAR mRNA in uPAR transfected HEK293T cells. Panels B to E show immunofluorescence staining of uPAR in HEK293T cells. Green staining represents P-cadherin, a member marker. uPAR is stained in red, and DAPI (4′,6-diamidino-2-phenylindole) is a blue-fluorescent DNA stain.

Figure S10. Immunostaining of murine liver tissue showing successful production of suPAR post-transfection

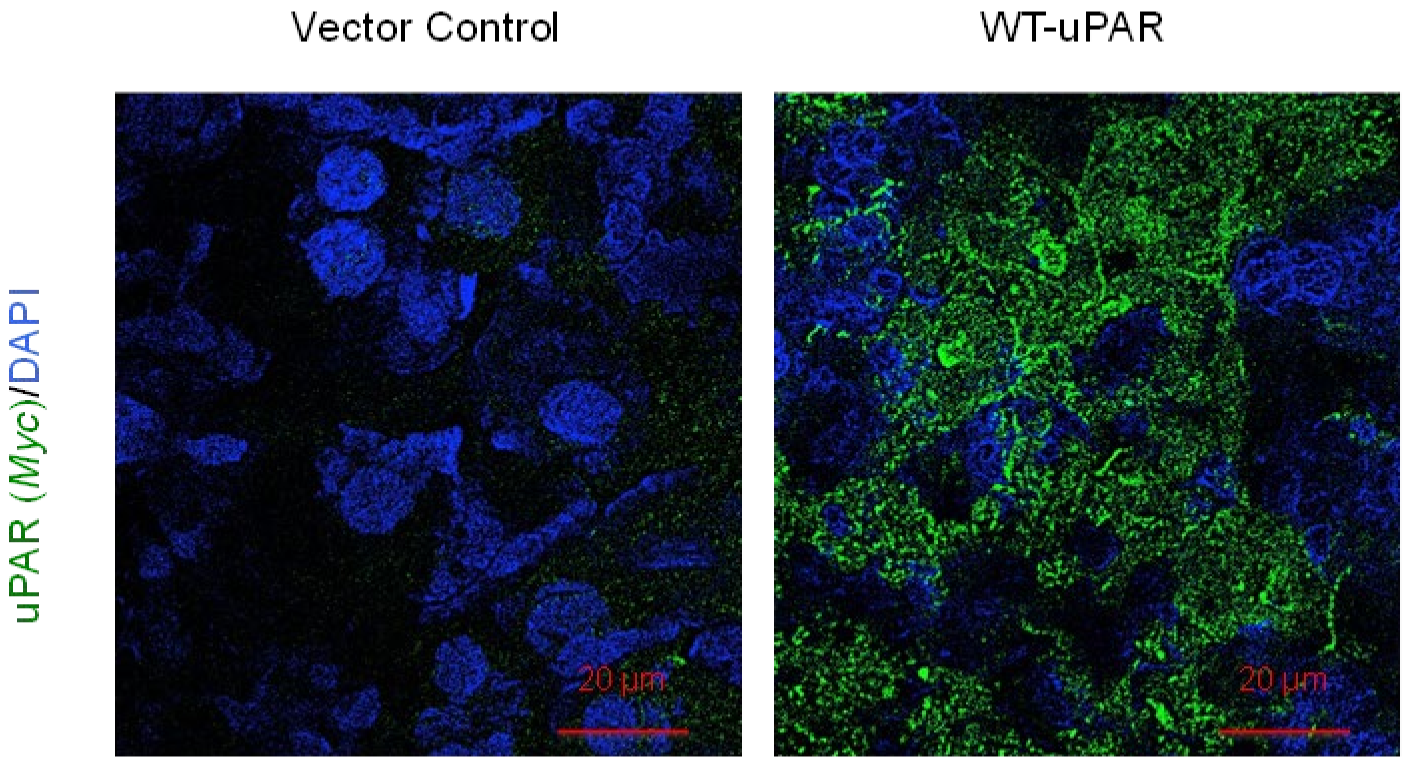

To confirm success of gene delivery in the liver and production of the protein post-hydrodynamic tail injection, we performed immunostaining of liver tissue using a Myc antibody detecting the c-terminal tag of uPAR-Myc fusion protein**.**

Figure S10. Mendelian randomization of genetically predicted suPAR by rs4760 with CVD in CARDIoGRAM C4D and the Million Veteran Program (MVP)

Disease	GWAS	Odds Ratio	[95% CI]	P
Coronary Artery Disease	CARDIOGRAM C4D	1.46	[1.14; 1.86]	0.002
Myocardial Infarction	CARDIOGRAM C4D	1.29	[0.98; 1.69]	0.069
Peripheral Artery Disease	Million Veterans Program	1.22	[1.00; 1.50]	0.046

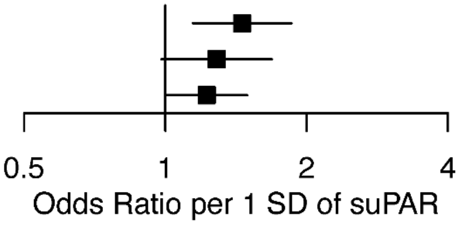

Figure S11. Mendelian randomization phenome-wide association of genetically-predicted suPAR by rs2302524 with CVD and rare damaging missense variants' impact on the odds of ischemic heart disease

Disease	Ncontrol	Ncase	Odds Ratio	[95% CI]	$P_{adjusted}$
Coronary Artery Disease	386561	22333	0.92	[0.81; 1.05]	0.50
Myocardial Infarction	391853	17041	0.85	[0.71; 1.01]	0.27
Peripheral Artery Disease	403178	5716	0.91	[0.70; 1.17]	0.60
Atrial Fibrillation	389031	19863	0.87	[0.76; 1.01]	0.27
Stroke	398090	10804	0.88	[0.73; 1.06]	0.44
Venous Thromboembolism	392652	15365	1.06	[0.91; 1.24]	0.60
Intracerebral Hemorrhage	407816	1078	1.24	[0.70; 2.19]	0.60
Hypertension	267082	141812	0.95	[0.89; 1.01]	0.37
Aortic Valve Stenosis	406359	2535	0.68	[0.46; 1.00]	0.27
Pulmonary Embolism	404322	4572	1.06	[0.80; 1.41]	0.78
Subarachnoid Hemorrhage	407717	1177	1.02	[0.59; 1.77]	0.94
Heart Failure	400799	8005	1.02	[0.82; 1.27]	0.92
Ischemic Stroke	403847	5047	0.90	[0.69; 1.18]	0.60

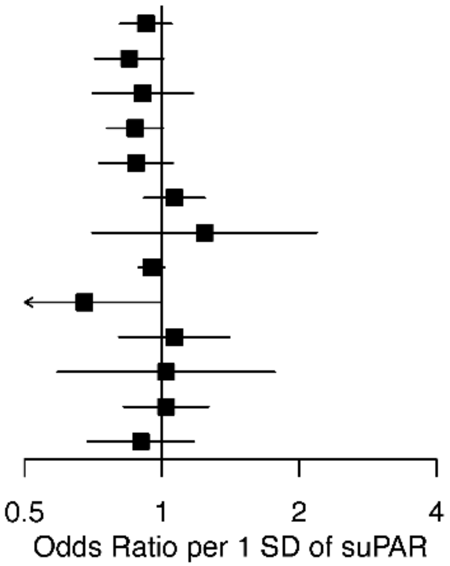

Causal effect of suPAR on 13 cardiovascular diseases by Mendelian randomization using missense variant rs2302524 as instrument. Effect estimates are provided per 1 standard deviation (SD) increase in suPAR levels. P values were adjusted using the false discovery rate method.

Figure S12. Association of suPAR with creatinine-derived eGFR and CKD as predicted by rs4760 *PLAUR* missense variant

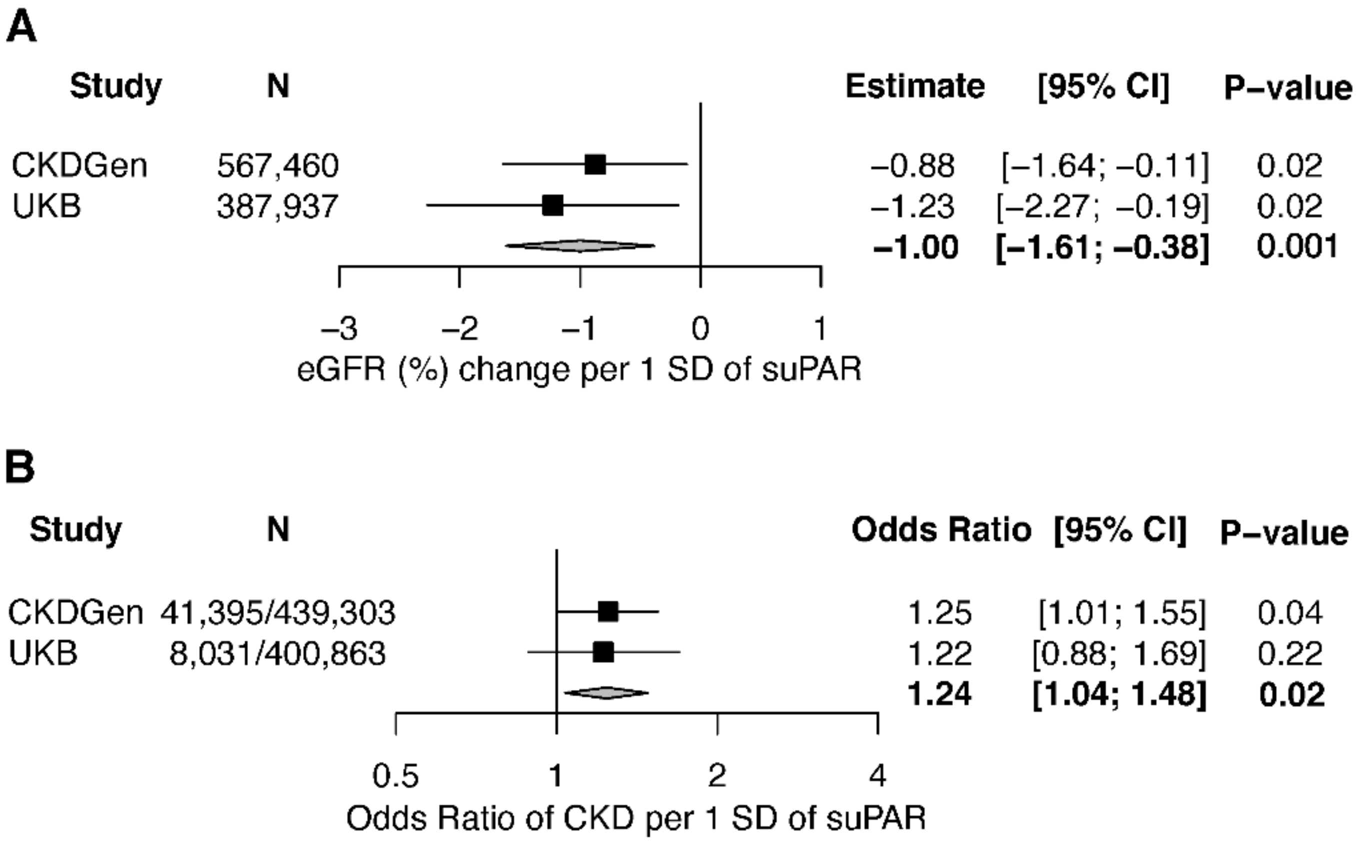

Mendelian randomization (MR) analysis for suPAR association with (**A**) estimated glomerular filtration rate (eGFR) and (**B**) chronic kidney disease (CKD) using summary data from the Chronic Kidney Disease (CKDGen) genome-wide association study and UK Biobank (UKB). Estimates per 1 standard deviation increments of suPAR instrumented by rs4760 were obtained using Wald ratio. Pooled effect estimates for both CKDGen and UKB were obtained using fixed-effects meta-analysis.

Figure S13. Cholesterol and suPAR levels in wild-type and transgenic mice prior to and 10 weeks after PSCK9-AAV transfection

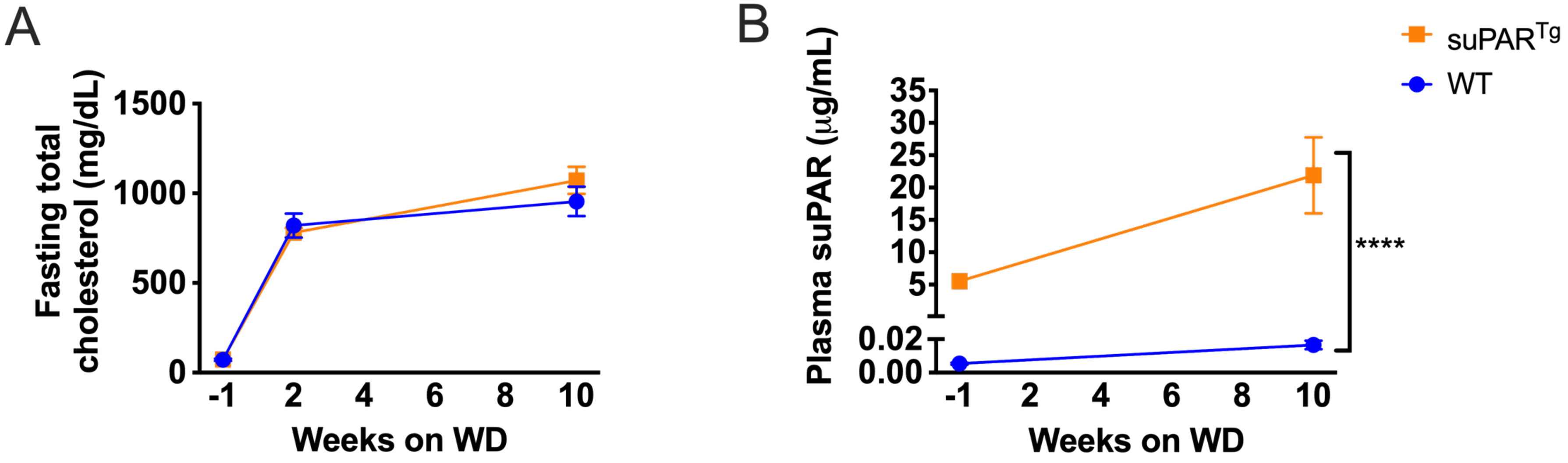

A, Fasting total cholesterol was quantified from plasma via colorimetric assay. Baseline measure was from immediately prior to PCSK9-AAV transfection followed by 1 week to rest mice, and western diet (WD) feeding began at week 0. n=18 WT and n=21 suPAR-Tg mice per group. 2-Way ANOVA. B, Plasma suPAR level assessed by ELISA at baseline and at the end of the study. 2-Way ANOVA. **** = P<0.0001.

Figure S14. Cholesterol, atherosclerotic lesion and necrotic sinus area in wild-type (male n=11, female n=7) and transgenic mice (male n=17, female n=4) over-expressing suPAR stratified by sex

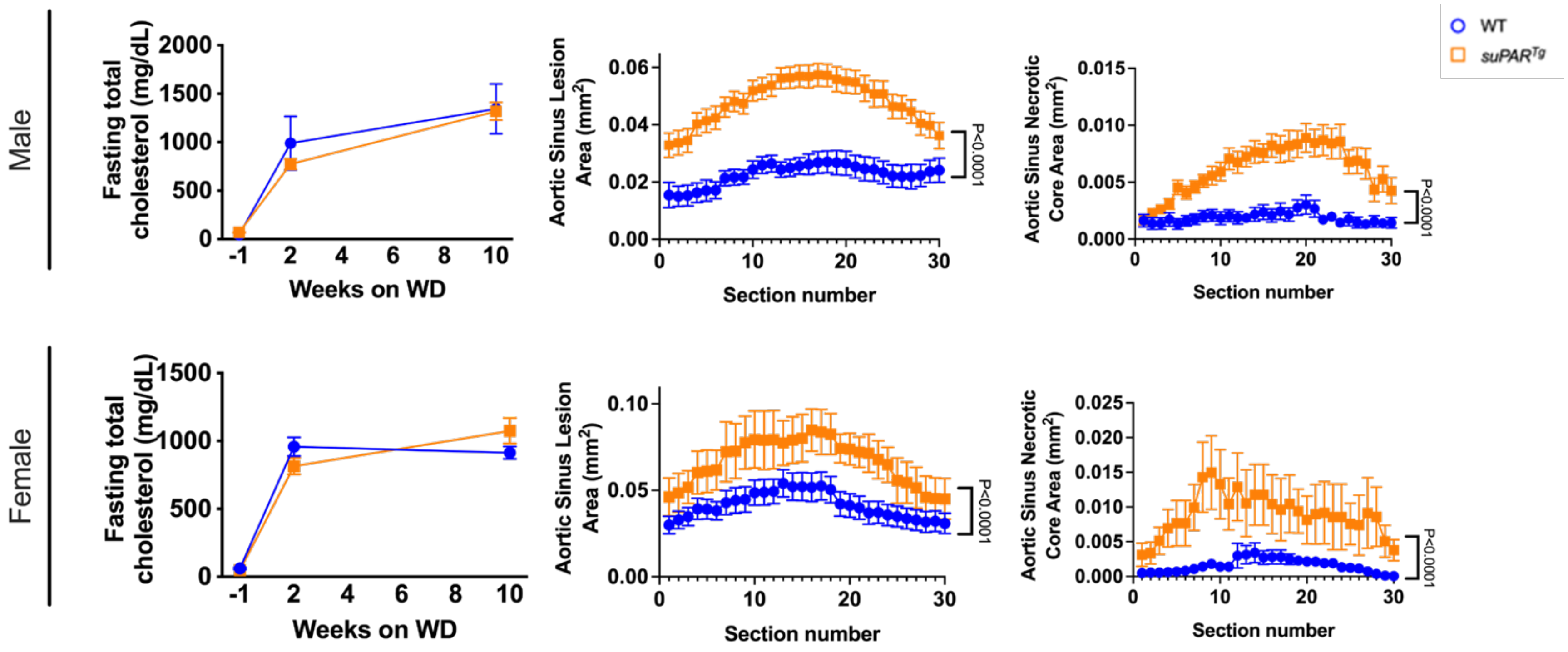

Fasting total cholesterol level of male WT (n=4), male suPARTg (n=9), female WT (n=5), female suPARTg (n=5) mice. Aortic sinus atherosclerotic plaque size and necrotic core size for male WT (n=11), male suPARTg (n=17), female WT (n=7), female suPARTg (n=4) mice.

Figure S15. SuPAR is detectable in both wild-type and suPAR-Tg atherosclerotic plaque

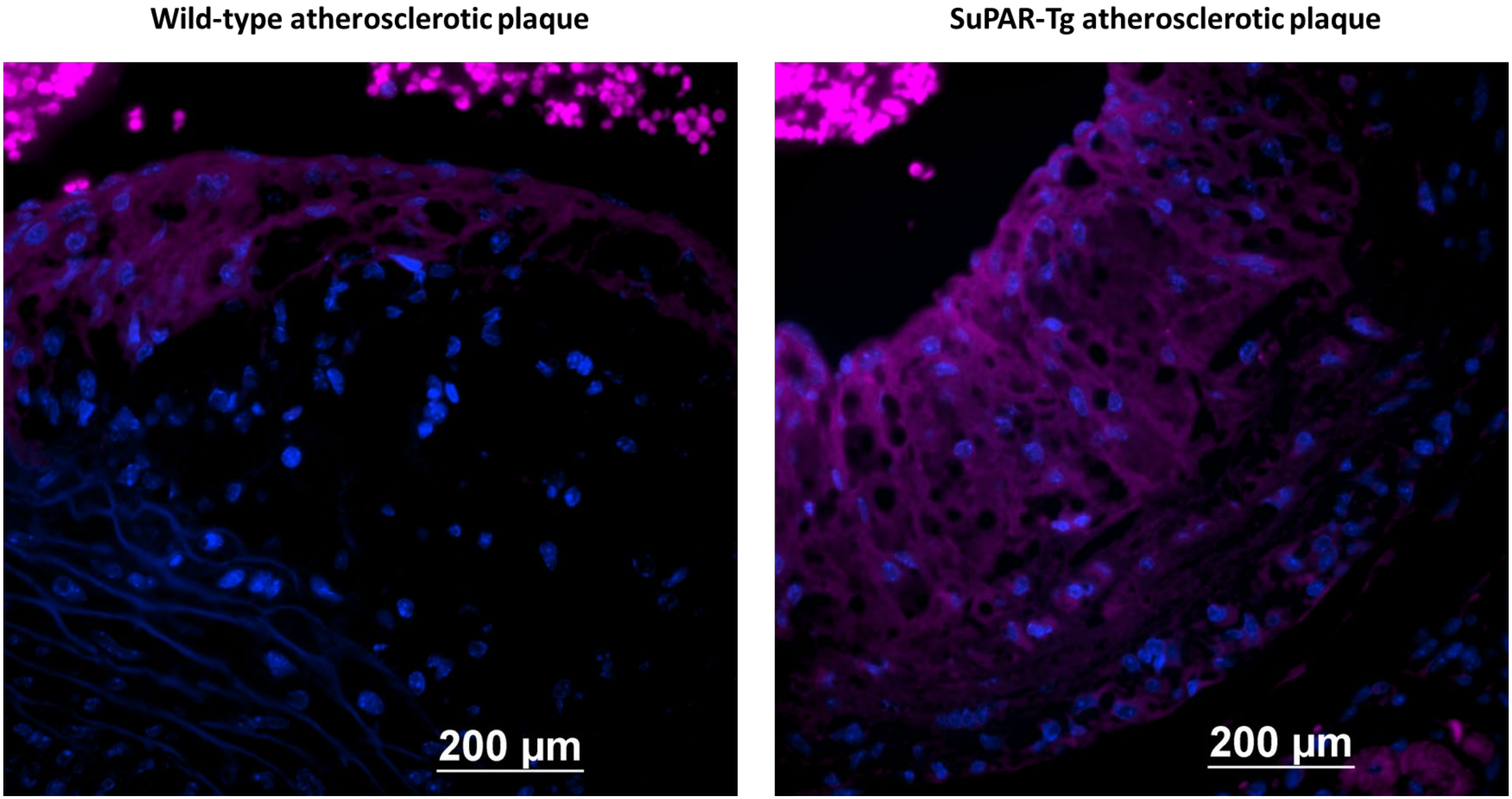

Representative images of immunofluorescence staining uPAR (magenta) in the atherosclerotic aortas of wild-type and suPAR-Tg mice, showing suPAR is detectable in the plaques of both strains, with increased deposition in suPAR-Tg plaque compared to wild-type. Nuclei are counter-stained blue with DAPI. Magnification: x60. Scale bar: 200µm.

Figure S16. Bone marrow-derived macrophages exhibit a pro-atherogenic phenotype compared to WT bone marrow-derived macrophages

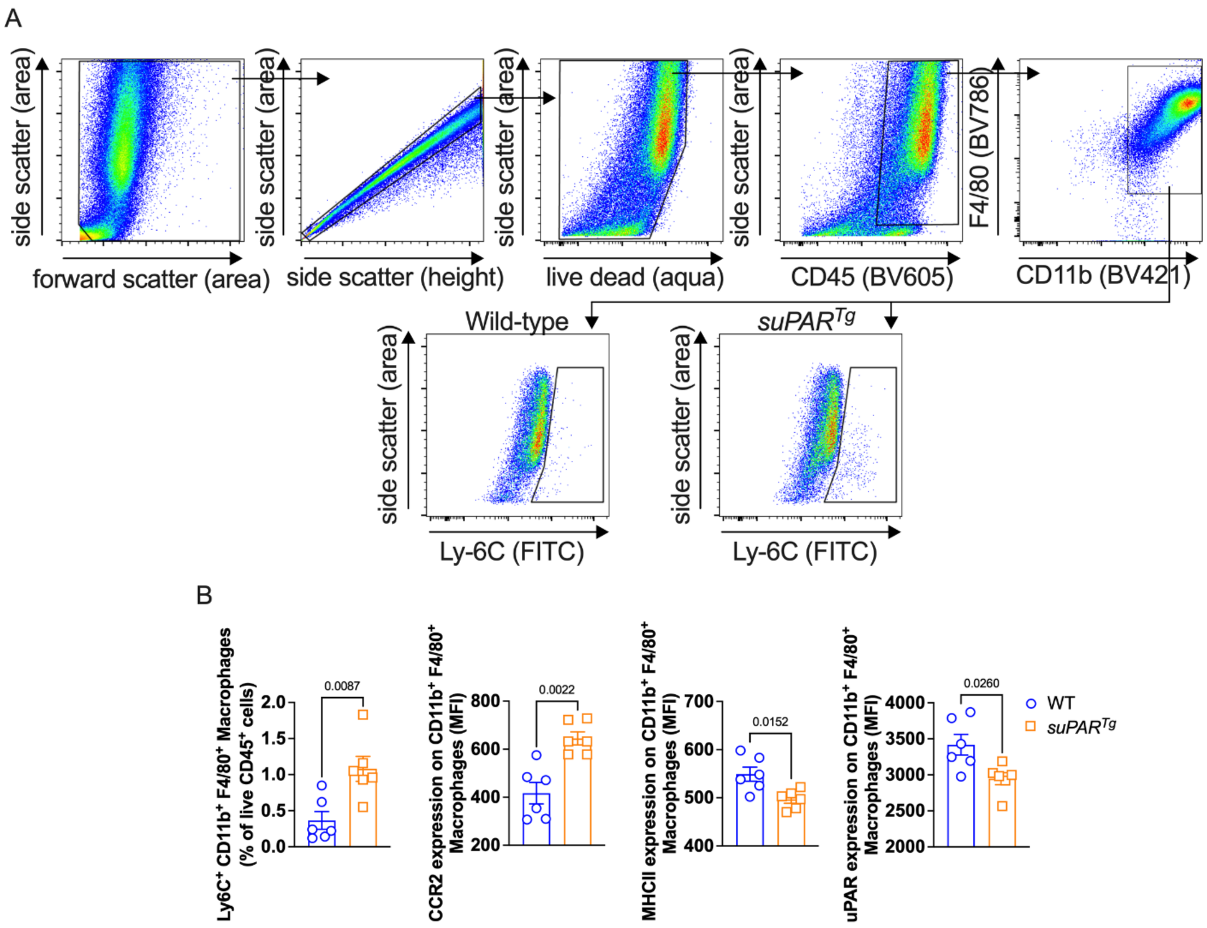

Panel A, More bone marrow-derived macrophages profiled by flow cytometry from suPARTg mice are Ly-6C+ compared to WT mice and have higher median fluorescence intensity of C-C Motif Chemokine Receptor 2 (CCR2). Bone marrow-derived macrophages profiled by flow cytometry from suPARTg mice have lower median fluorescence intensity expression of major histocompatibility complex II (MHCII) and urokinase plasminogen activator receptor (uPAR). n=6 per group. Mann-Whitney U test. Each point is a biological replicate.

Figure S17. QQ plots for genome-wide associations with suPAR

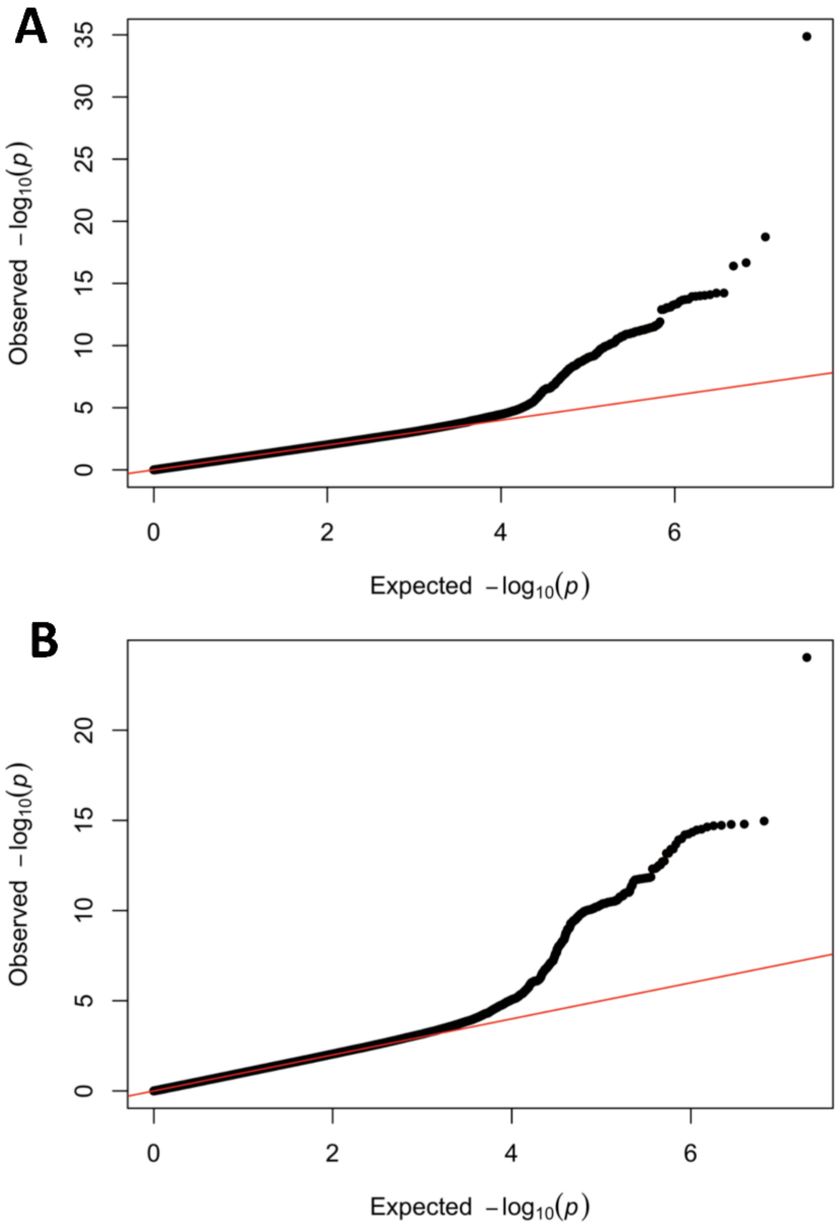

Quantile-Quantile plots for observed versus expected P-values in the multi-ancestry genome-wide meta-analysis (A) or European ancestry meta-analysis (B). The genomic control lambdas were 1.01 and 1.02 in the multi-ancestry and European ancestry analyses, respectively, indicating no evidence of population stratification.

Figure S18. Chromatogram by Sanger sequencing of *PLAUR* reference allele and variants

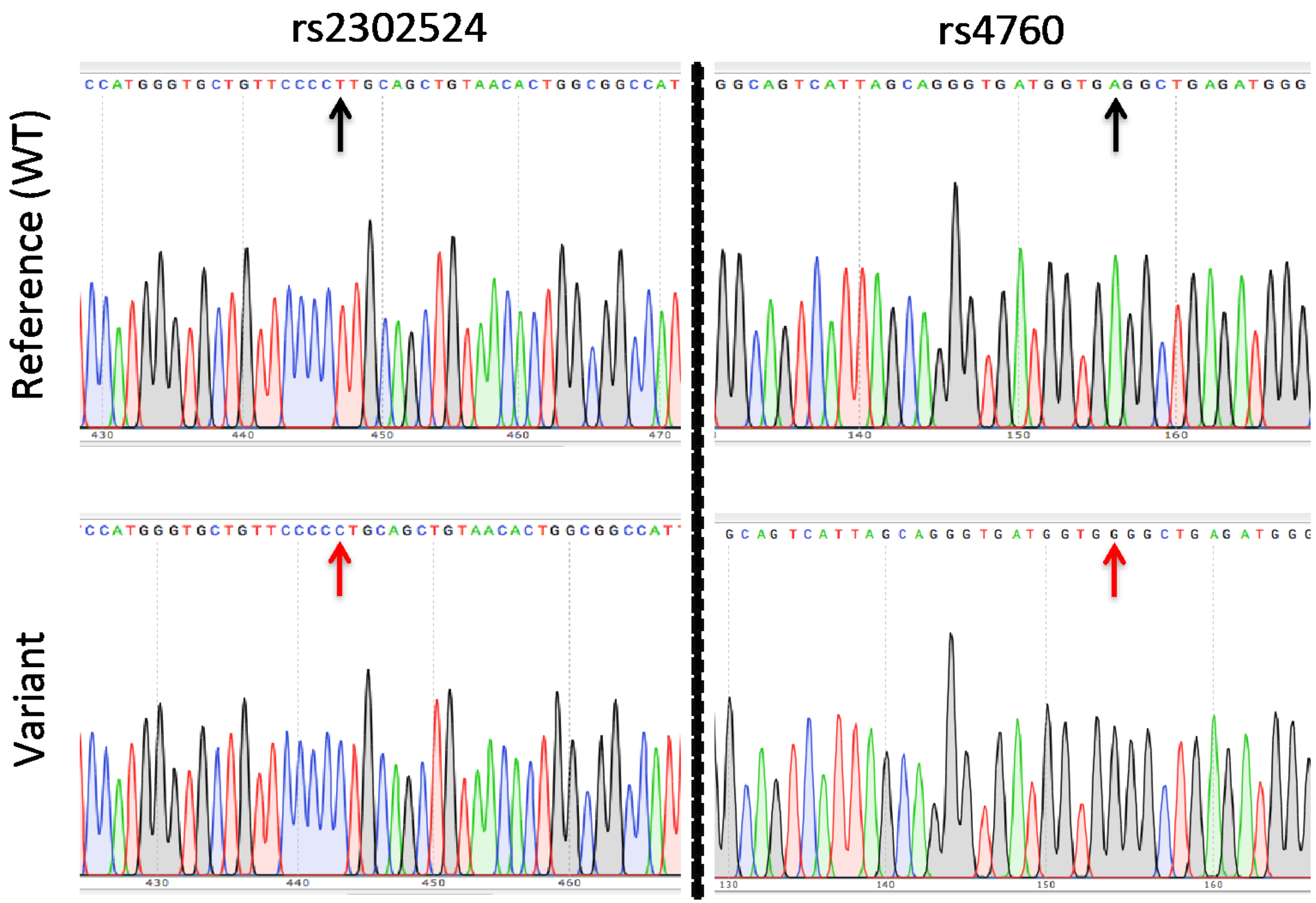

The wild-type PLAUR (reference, Gene accession #NM_002659) was cloned into a pCMV6-entry vector (Origene). The *PLAUR* variants rs2302524 and rs4760 were created using the GeneArt site directed mutagenesis system (Thermo Scientific). Black arrow indicates reference nucleotide. Red arrow indicates variant nucleotide.

Table S1. Baseline characteristics of MESA cohort by suPAR categories

	Total (n=5,406)	0-2.0 ng/mL (n=1456)	2.0-2.5 ng/mL (n=1357)	2.5-3.0 ng/mL (n=1099)	>3.0 ng/mL (n=1494)	P-value
**Demographics**						
Age (years), mean (SD)	63 (10)	59 (9)	62 (10)	65 (10)	65 (11)	<0.001
Male, n (%)	2620 (48.5)	858 (58.9)	644 (47.5)	502 (45.7)	616 (41.2)	<0.001
Race/ethnicity, n (%)						<0.001
African American	1464 (27.1)	421 (28.9)	328 (24.2)	270 (24.6)	445 (29.8)	
Chinese American	667 (12.3)	321 (22.0)	153 (11.3)	89 (8.1)	104 (7.0)	
Hispanic	1221 (22.6)	205 (14.1)	341 (25.1)	293 (26.7)	382 (25.6)	
White	2054 (38.0)	509 (35.0)	535 (39.4)	447 (40.7)	563 (37.7)	
**Cardiovascular disease risk factors**						
Body mass index, mean (SD)	28 (5)	27 (5)	28 (5)	29 (6)	29 (6)	<0.001
Ever smoker, n (%)	2664 (49.5)	656 (45.3)	651 (48.1)	560 (51.2)	797 (53.5)	<0.001
Diabetes, n (%)	685 (12.7)	121 (8.3)	149 (11.0)	135 (12.3)	280 (18.7)	<0.001
Hypertension, n (%)	2448 (45.3)	536 (36.8)	563 (41.5)	499 (45.4)	850 (56.9)	<0.001
**Laboratory values, median (IQR)**						
eGFR, mL/min	77.3 (66.2, 88.2)	82.4 (71.8, 92.6)	78.3 (67.9, 88.2)	74.9 (64.4, 86.2)	72.0 (60.4, 85.7)	<0.001
Total cholesterol, mg/dL	192 (170, 216)	193 (173, 215)	195 (172, 217)	192 (171, 216)	188 (165, 213)	<0.001
High-density cholesterol, mg/dL	48 (40, 59)	49 (41, 60)	50 (41, 60)	48 (40, 59)	46 (39, 56)	<0.001
Low-density cholesterol, mg/dL	116 (96, 136)	118 (98, 136)	117 (97, 136)	116 (95, 135)	112 (92, 136)	<0.001
suPAR, ng/mL	2.5 (2.0, 3.1)	1.7 (1.5, 1.9)	2.3 (2.1, 2.4)	2.7 (2.6, 2.9)	3.7 (3.3, 4.3)	<0.001
Coronary artery calcium, AU	1.9 (0, 97.9)	0 (0, 47.2)	0 (0, 82.6)	5.5 (0, 148.0)	12.2 (0, 146.4)	<0.001

Table S2. Adjusted association between suPAR level and baseline CAC score at baseline in MESA

	ß[a] (95% CI)	P-value
Age, years	10.4 (9.1, 11.7)	<0.001
Race/ethnicity, n (%)		
White	1.0 [reference]	
African American	-96.7 (-124.9, -68.4)	<0.001
Hispanic	-67.9 (-97.4, -38.4)	<0.001
Chinese American	-89.9 (-127.3, -52.5)	<0.001
Male	153.2 (128.5, 178.0)	<0.001
Body mass index, kg/m^2	2.8 (0.5, 5.2)	0.018
Ever smoker	22.7 (0.3, 45.0)	0.047
Diabetes	86.1 (52.0, 120.1)	<0.001
Hypertension	65.6 (41.7, 89.3)	<0.001
eGFR, per 5 mL/min	5.4 (-2.5, 13.3)	0.18
High-density cholesterol, per 5 mg/dL	2.1 (-6.3, 10.5)	0.63
Low-density cholesterol, per 10mg/dL	1.3 (-2.2, 4.7)	0.47
C-reactive protein, per mg/L	-9.1 (0.3, 50.0)	0.015
**SuPAR**	**28.7 (8.0, 49.5)**	**0.007**

[a]Beta corresponds to each 2-fold difference in suPAR levels
Abbreviations: CAC, coronary artery calcium; CI, confidence interval; suPAR, soluble urokinase plasminogen activator receptor

Table S3. Longitudinal association between baseline suPAR levels and CAC score during follow-up in MESA

	ß (95% CI)	P-value
Age, years	0.9 (0.4, 1.5)	<0.001
Race/ethnicity, n (%)		
White	1.0 [reference]	
African American	-6.1 (-18.5, 6.3)	0.33
Hispanic	-9.2 (-22.6, 4.1)	0.17
Chinese American	-11.6 (-23.7, 0.5)	0.06
Male	14.6 (4.8, 24.4)	0.003
Body mass index, kg/m^2	0.6 (-0.6, 1.7)	0.34
Ever smoker	3.9 (-4.8, 12.5)	0.38
Diabetes	19.3 (11.9, 26.7)	<0.001
Hypertension	19.3 (10.3, 28.4)	<0.001
eGFR, per 5 mL/min	-1.6 (-5.7, 2.5)	0.45
High-density cholesterol, per 5 mg/dL	-1.5 (-4.8, 1.8)	0.37
Low-density cholesterol, per 10mg/dL	0.01 (-1.4, 1.4)	0.98
Baseline CAC	1.3 (1.2, 1.3)	<0.001
Follow-up, years	15.6 (5.2, 25.9)	0.005
C-reactive protein, per mg/L	1.62 (-1.36, 4.61)	0.29
SuPAR, per 100% increase[a]	-24.4 (-38.2, -10.7)	<0.001
**SuPAR*follow-up[b]**	**15.0 (6.6, 23.4)**	**<0.001**

[a]Beta corresponds to each 2-fold difference in suPAR levels
[b]Beta corresponds to yearly increase in the CAC score per 2-fold difference in suPAR levels

Table S4. Multivariable survival analysis for baseline suPAR levels and CVD events in MESA

	HR (95% CI)	P-value
Age, years	1.06 (1.05, 1.07)	<0.001
Race/ethnicity, n (%)		
White	1.0 [reference]	
African American	0.82 (0.68, 0.99)	0.034
Hispanic	0.96 (0.80, 1.16)	0.66
Chinese American	0.87 (0.67, 1.12)	0.27
Male	1.82 (1.54, 2.14)	<0.001
Body mass index, per 5 kg/m^2	1.01 (0.93, 1.09)	0.86
Ever smoker	1.12 (1.01, 1.11)	0.024
Diabetes	1.70 (1.42, 2.03)	<0.001
Hypertension	1.83 (1.56, 2.14)	<0.001
eGFR, per 5 mL/min	1.01 (0.96, 1.06)	0.74
High-density cholesterol, per 5 mg/dL	0.94 (0.88, 0.99)	0.032
Low-density cholesterol, per 10mg/dL	1.03 (1.01, 1.06)	0.004
C-reactive protein, per mg/L	1.06 (1.01, 1.11)	0.024
**SuPAR, log-base 2**	**1.46 (1.29, 1.65)**	**<0.001**
**SuPAR, categorical**		**<0.001**
0-2.0 ng/mL	1.0 [reference]	
2.0-2.5 ng/mL	1.11 (0.88, 1.39)	0.37
2.5-3.0 ng/mL	1.38 (1.09, 1.74)	0.006
>3.0 ng/mL	1.77 (1.42, 2.19)	<0.001

Number of cardiovascular disease (CVD) events was 594. A CVD event was defined as the composite of myocardial infarction, resuscitated cardiac arrest, angina, revascularization, stroke (excluding transient ischemic attack), or death due to CVD.

Table S5. Multivariable survival analysis for baseline suPAR levels and CVD events in MESA with time-varying eGFR

	**HR (95% CI)**	**P-value**
Age, years	1.06 (1.05, 1.07)	<0.001
Race/ethnicity, n (%)		
White	1.0 [reference]	
African American	0.83 (0.70, 1.00)	0.05
Hispanic	0.98 (0.81, 1.17)	0.82
Chinese American	0.87 (0.68, 1.12)	0.28
Male	1.83 (1.55, 2.16)	<0.001
Body mass index, per 5 kg/m^2	1.00 (0.93, 1.09)	0.89
Ever smoker	1.13 (0.97, 1.30)	0.11
Diabetes	1.71 (1.43, 2.04)	<0.001
Hypertension	1.80 (1.54, 2.11)	<0.001
eGFR, per 5 mL/min	0.97 (0.95, 1.00)	0.08
High-density cholesterol, per 5 mg/dL	0.94 (0.89, 1.00)	0.037
Low-density cholesterol, per 10mg/dL	1.03 (1.01, 1.06)	0.005
C-reactive protein, per mg/L	1.06 (1.01, 1.11)	0.026
**SuPAR, log-base 2**	**1.43 (1.26, 1.61)**	**<0.001**
**SuPAR, categorical**		**<0.001**
0-2.0 ng/mL	1.0 [reference]	
2.0-2.5 ng/mL	1.10 (0.88, 1.38)	0.41
2.5-3.0 ng/mL	1.36 (1.08, 1.71)	0.009
>3.0 ng/mL	1.71 (1.38, 2.12)	<0.001

Number of cardiovascular disease (CVD) events was 594. A CVD event was defined as the composite of myocardial infarction, resuscitated cardiac arrest, angina, revascularization, stroke (excluding transient ischemic attack), or death due to CVD.

Table S6. Multivariable survival analysis for baseline suPAR levels and CVD events in MESA with high-sensitivity troponin T and NT-proBNP

	HR (95% CI)	P-value
Age, years	1.04 (1.03, 1.05)	<0.001
Race/ethnicity, n (%)		
White	1.0 [reference]	
African American	0.91 (0.74, 1.11)	0.39
Hispanic	1.02 (0.78, 1.34)	0.91
Chinese American	0.99 (0.81, 1.21)	0.86
Male	1.43 (1.17, 1.74)	0.001
Body mass index, per 5 kg/m^2	0.94 (0.87, 1.02)	0.15
Ever smoker	1.10 (0.95, 1.28)	0.20
Diabetes	1.56 (1.28, 1.90)	<0.001
Hypertension	1.70 (1.45, 2.00)	<0.001
eGFR, per 5 mL/min	1.05 (1.00, 1.10)	0.06
High-density cholesterol, per 5 mg/dL	0.89 (0.83, 0.95)	0.002
Low-density cholesterol, per 10mg/dL	1.04 (1.02, 1.06)	0.001
C-reactive protein, per mg/L	1.07 (1.02, 1.12)	0.005
High-sensitivity troponin T (ng/L)	1.48 (1.30, 1.70)	<0.001
NT-proBNP (pg/mL)	1.21 (1.12, 1.31)	<0.001
**SuPAR, log-base 2**	**1.37 (1.21, 1.56)**	**<0.001**

A CVD event was defined as the composite of myocardial infarction, resuscitated cardiac arrest, angina, revascularization, stroke (excluding transient ischemic attack), or death due to CVD

Table S7. Top variants from genome-wide association analysis of suPAR in participants with European ancestry

							TSS, GABC, MESA, MDC (n=12,937)			DBDS (n=12,177)		
**SNP**	**Chromosome**	**Position**	**Locus**	**EA**	**OA**	**EAF**	**Effect**	**SE**	**P-value**	**Effect**	**SE**	**P-value**
rs12077698	1	38088049	*POU3F1*	C	G	0.08	-0.23	0.04	$2×10^{-10}$	0.00	0.04	0.96
rs7595388	2	159899834	*LY75*	A	G	0.34	-0.10	0.01	$4×10^{-12}$	-0.10	0.01	$5 × 10^{-14}$
rs56172805	3	98907345	*DCBLD2*	A	G	0.05	-0.22	0.03	$3×10^{-11}$	-0.20	0.03	$7 × 10^{-10}$
rs79493037	6	31438048	*MICA*	G	C	0.13	-0.18	0.03	$6×10^{-09}$	-0.06	0.03	0.02
rs2633313	10	73924107	*PLAU*	C	T	0.46	-0.12	0.01	$1×10^{-15}$	-0.11	0.01	$1 × 10^{-18}$
rs240559	11	126321217	*ST3GAL4*	T	C	0.21	0.09	0.02	$3×10^{-08}$	0.05	0.01	0.001
rs3967200	11	126362490	*ST3GAL4*	T	C	0.17	-0.13	0.02	$1×10^{-09}$	-0.14	0.02	$2 × 10^{-14}$
rs535064984	17	7116978	*ASGR2*	C	T	0.004	-0.93	0.17	$4×10^{-08}$	0.54	0.09	$4 × 10^{-09}$
rs55714927	17	7176997	*ASGR1*	T	C	0.16	0.12	0.02	$2×10^{-11}$	0.12	0.02	$2 × 10^{-14}$
rs4251824	19	43666441	*PLAUR*	T	C	0.04	-0.24	0.04	$1×10^{-11}$	-0.24	0.03	$4 × 10^{-17}$
rs2302524	19	43652320	*PLAUR*	C	T	0.17	0.20	0.02	$9×10^{-25}$	0.10	0.02	$3 × 10^{-8}$
rs4760	19	43648948	*PLAUR*	G	A	0.10	0.13	0.02	$2×10^{-10}$	0.08	0.02	$6 × 10^{-6}$

Abbreviations: GABC: Genes of Blood-Clotting Cohort; DBDS: Danish Blood Donor Study; EA, Effect Allele; EAF, Effect Allele Frequency; MESA: Multi-Ethnic Study of Atherosclerosis; MDC: Malmo Diet and Cancer study; OA, Other Allele; SE, Standard Error; SNP, Single Nucleotide Polymorphism; TSS: Trinity Student Study.

Table S8. Finemapped signals of the *PLAUR* locus using SuSie.

rsid	POS37	Allele 1	Allele 2	Effect	Standard error	P-value	Posterior Inclusion Probability (PIP)	Credible set
rs2302524	44156472	T	C	-0.1959	0.0191	9.31×10^{-25}	1	1
rs4760	44153100	A	G	-0.1316	0.0208	2.38×10^{-10}	0.999998049	2
rs4251824	44170593	T	C	-0.2401	0.0354	1.21×10^{-11}	0.487264651	3
rs117564136	44177700	T	C	0.1374	0.0259	1.08×10^{-07}	0.83801097	4
rs400058	44141015	T	C	0.0362	0.0163	0.026	0.999543437	4

Table S9. UK Biobank Disease Phenotype Definitions

Disease phenotype	Definition
Aortic valve stenosis	Self-reported history of aortic stenosis during verbal interview with trained nurse; or hospitalization with or death due to ICD-10 code for rheumatic aortic stenosis (I06.0, I06.2) or nonrheumatic aortic stenosis (I35.0, I35.2)
Atrial fibrillation or flutter	Self-reported history of atrial fibrillation, atrial flutter, or cardioversion during verbal interview with trained nurse; or hospitalization with or death due to ICD-10 code for atrial fibrillation or atrial flutter (I48); or hospitalization with ICD-9 code for atrial fibrillation or atrial flutter (4273); or hospitalization with OPCS-4 code for percutaneous transluminal ablation (K57.1, K 62.1, K62.2, K62.3, K62.4)
Coronary artery disease	Self-reported history of myocardial infarction, coronary artery bypass grafting, coronary artery angioplasty or triple heart bypass during verbal interview with trained nurse; or hospitalization for or death due to ICD-10 code for acute or subsequent myocardial infarction (I21, I22, I23, I24.1, I25.2); or hospitalization due to ICD-9 code for myocardial infarction (410, 411, 412); or hospitalization due to OPCS-4 code for coronary artery bypass grafting (K40, K41, K44, K45, K46), coronary endarterectomy (K47.1), or coronary angioplasty ± stenting (K49, K50.2, K75)
Heart failure	Self-reported history of heart failure or cardiomyopathy during verbal interview with trained nurse; or hospitalization for or death due to ICD-10 code for hypertensive heart disease, cardiomyopathy or heart failure (I11.0, I13.0, I13.2, I25.5, I42.0, I42.5, I42.8, I42.9, I50.0, I50.1, I50.9); or hospitalization due to ICD-9 code for heart failure or other primary cardiomyopathies (4254, 4280, 4281, 4289); excluding individuals with history of hypertrophic cardiomyopathy during verbal interview with trained nurse, or hospitalization for or death due to ICD-10 code for hypertrophic cardiomyopathy (I42.1, I42.2)
Hypertension	Self-reported history of hypertension, essential hypertension or high blood pressure during verbal interview with trained nurse; or hospitalization with or death due to ICD-10 code for essential hypertension, hypertensive heart disease, hypertensive renal disease, or secondary hypertension (I10, I11, I12, I13, I15); or hospitalization with ICD-9 code for essential hypertension, hypertensive heart disease, hypertensive renal disease, or secondary hypertension (401, 402, 403, 404, 405)
Intracerebral hemorrhage	Self-reported history of brain hemorrhage during verbal interview with trained nurse; or hospitalization with or death due to ICD-10 code for nontraumatic intracerebral hemorrhage (I61); or hospitalization with or death due to ICD-9 code for intracerebral hemorrhage (431), as adjudicated centrally by the UK Biobank
Ischemic stroke	Self-reported history of ischemic stroke during verbal interview with trained nurse; or hospitalization with or death due to ICD-10 code for cerebral infarction, or unspecified stroke (I63, 64); or hospitalization with or death due to ICD-9 code for occlusion of cerebral arteries or acute cerebrovascular disease (434, 436), as adjudicated centrally by the UK Biobank
Myocardial infarction	Self-reported history of myocardial infarction or hospitalization or death due to ICD-10 code for acute or subsequent myocardial infarction (I21, I22, I23, I24.1, I25.2); or hospitalization due to ICD-9 code for myocardial infarction (410, 411, 412)
Peripheral artery disease	Self-reported history of peripheral vascular disease, arterial embolism, intermittent claudication, leg artery bypass, leg artery angioplasty, or leg amputation during verbal interview with trained nurse; or hospitalization with or death due to ICD-10 code for atherosclerosis of (non-coronary) arteries or peripheral vascular disease (I70.0, I70.00, I70.01, I70.2, I70.20, I70.21, I70.8, I70.80, I70.9, I70.90, I73.8 or I73.9); or hospitalization with ICD-9 code for atherosclerosis of arteries or peripheral vascular disease (4400, 4402, 4438, 4439); or hospitalization with OPCS-4 coded procedure for leg amputation, or leg artery procedure such as bypass, stent or angioplasty (X09.3-09.5, L21.6, L51.3, L51.6, L51.8, L52.1, L52.2, L54.1, L54.4, L54.8, L59.1-L59.8, L60.1, L60.2, L63.1, L63.5, L63.9, L66.7)

Pulmonary embolism	Self-reported history of pulmonary embolism during verbal interview with trained nurse; or hospitalization with or death due to ICD-10 code for pulmonary embolism (I26); or hospitalization with ICD-9 code for pulmonary embolism (4151)
Stroke	Self-reported history of stroke during verbal interview with trained nurse; or hospitalization with or death due to ICD-10 code for nontraumatic subarachnoid hemorrhage, nontraumatic intracerebral hemorrhage, cerebral infarction, or unspecified stroke (I60-64); or hospitalization with or death due to ICD-9 code for subarachnoid hemorrhage, intracerebral hemorrhage, occlusion of cerebral arteries, or acute cerebrovascular disease (430, 431, 434, 436), as adjudicated centrally by the UK Biobank (http://biobank.ctsu.ox.ac.uk/crystal/refer.cgi?id=462)
Subarachnoid hemorrhage	Self-reported history of subarachnoid hemorrhage during verbal interview with trained nurse; or hospitalization with or death due to ICD-10 code for nontraumatic subarachnoid hemorrhage (I60); or hospitalization with or death due to ICD-9 code for subarachnoid hemorrhage (430), as adjudicated centrally by the UK Biobank
Venous thromboembolism	Self-reported history of venous thromboembolic disease, pulmonary embolism or deep venous thrombosis during verbal interview with trained nurse; or hospitalization with or death due to ICD-10 code for pulmonary embolism (I26), phlebitis or thrombophlebitis (I80.0-I80.3, I80.8-I80.9), portal vein thrombosis (I81), Budd-Chiari syndrome (I82.0), or other coagulation defects (D68); or hospitalization with ICD-9 code for pulmonary embolism or phlebitis/thrombophlebitis (4151, 4511); or hospitalization with OPCS-4 code for insertion of inferior vena cava filter or open thrombectomy of lower extremity vein (L79.1, L90.2)

REFERENCES

1. Liang OD, Chavakis T, Kanse SM, Preissner KT. Ligand binding regions in the receptor for urokinase-type plasminogen activator. J Biol Chem 2001;276:28946-53.
